# Synergistic action of two radical SAM enzymes in the biosynthesis of thuricin CD, a two-component sactibiotic†

**DOI:** 10.1039/d5sc01546d

**Published:** 2025-05-08

**Authors:** Yifei Jia, Yuanjun Han, Xuxue Liu, Qi Zhang

**Affiliations:** a Department of Chemistry, Fudan University No. 2005 Songhu Road Shanghai 200433 China qizhang@sioc.ac.cn; b National Engineering Research Center for Carbohydrate Synthesis, College of Chemistry and Materials, Jiangxi Normal University No. 99 Ziyang Avenue Nanchang 330022 China

## Abstract

Thuricin CD, composed of two ribosomally derived peptides trnα and trnβ, is a distinct two-component sactipeptide antibiotic known for its potent narrow spectrum antibacterial activity against several strains including *Clostridioides difficile*. Despite its early discovery, how the characteristic thioether crosslinks are installed on thuricin CD remained largely elusive. In this report, we demonstrate that neither of the two radical *S*-adenosylmethionine (rSAM) enzymes, TrnC and TrnD, can effectively modify the precursors individually. Instead, TrnC and TrnD form a tight complex and collaboratively catalyze thioether crosslinking on the two precursor peptides TrnA and TrnB. Although both TrnC and TrnD are active rSAM enzymes, only the rSAM activity of TrnC is strictly essential for thioether crosslinking, demonstrating a unique enzyme synergy in the biosynthesis of two-component antibiotics. We also generate an active thuricin CD variant by a procedure involving coexpression followed by *in vitro* proteolysis.

## Abstract 2

Radical SAM enzymes TrnC and TrnD collaborate to install thioether crosslinks in thuricin CD, with TrnC as the key catalyst—unveiling a rare two-component antibiotic biosynthesis.

## Graphical abstract

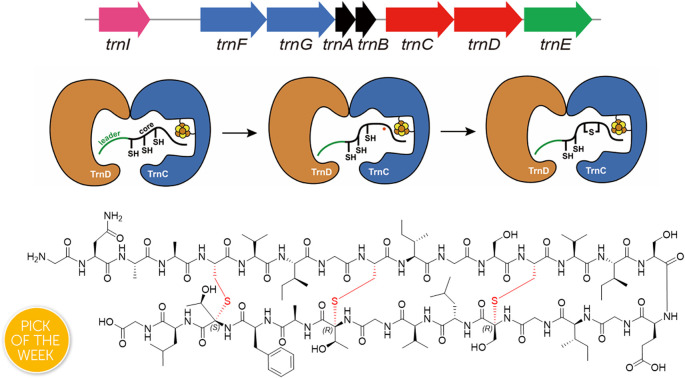

## Introduction

Combination therapy is a common approach to enhance antibiotic effectiveness, and such a strategy has long been employed by nature in the secretion of two-component antibiotics. A notable group of two-component antibiotics comprises the class IIb bacteriocins usually derived from lactic acid bacteria, which commonly adopt a helix–helix structure to result in the formation of pores in the cell membrane.^1,2^ Another significant source of two-component antibiotics is lanthipeptides, defined as lanthionine-containing peptides,^3,4^ which represent the largest and most extensively characterized family within the ribosomally synthesized and posttranslationally modified peptide (RiPP) natural product superfamily.^5–7^ Thus far, two-component lanthipeptides all belong to the class II lanthipeptide subfamily, whose biosynthesis involves LanM-type modification enzymes, a group of bifunctional proteins catalyzing both dehydration and lanthionine formation.^3,4^ Two-component lanthipeptides can be produced by a single LanM enzyme, as exemplified by cytolysin.^8^ More frequently, two LanM enzymes are encoded in the gene cluster, each individually responsible for modifying a precursor peptide, as have been found in haloduracin, lichenicidin, and lacticin 3147.^2–4^

**Fig. 1:**
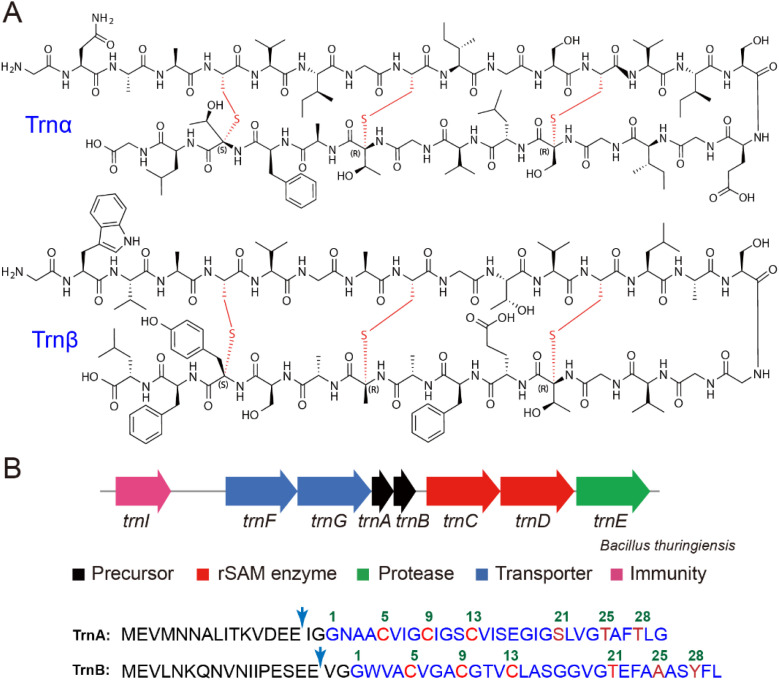
Thuricin CD produced by *Bacillus thuringiensis*. (A) The chemical structure of trnα and trnβ. (B) The biosynthetic gene cluster (BGC) of thuricin CD, showing the gene organization and the sequence of the two precursor peptides TrnA and TrnB. The leaders and cores are shown in black and blue, respectively, and the residues involved in thioether crosslinks are shown in red (Cys) and dark red (other residues), respectively. The GluC proteolytic cleavage site utilized in this study for producing a thuricin CD variant are denoted by blue arrows.

Thuricin CD produced by *Bacillus thuringiensis* represents a unique example of a two-component antibiotic (Fig. 1A).^9^ Thuricin CD comprises two peptides, trnα and trnβ, both belonging to the sactipeptide family. Sactipeptides are a group of RiPP natural products containing sactionine residues, which feature a unique sulfur-to-alpha-carbon thioether linkage, and sactipeptide members possessing antibiotic activities are also termed sactibiotic.^5,6,10–12^ Thuricin CD is the only known two-component sactibiotic and exhibits potent activity against various Clostridia species, particularly the clinically relevant pathogen *Clostridioides difficile*. Its bactericidal mechanism involves targeting the cell membrane, disrupting membrane potential, and ultimately leading to cell collapse. These properties make thuricin CD a promising candidate for therapeutic and biotechnological applications.^13–18^ Trnα and trnβ feature a hairpin-like structure containing three nested thioether crosslinks, with Cys residues in the N-termini (Fig. 1A). Such a nested ring topology is commonly found in sactipeptides such as subtilosin A,^19–21^ thurincin H,^22–24^ thuricin Z (huazacin),^25,26^ and hyicin 4244,^27^ whereas varied ring topologies are also observed (*e.g.* sporulation killing factor SKF,^28–30^ ruminococcin,^31–34^ streptosactin,^35^ and enteropeptins^36^).

The biosynthetic gene cluster responsible for thuricin CD production encodes two radical *S*-adenosylmethionine (radical SAM, rSAM) enzymes, TrnC and TrnD, along with the two precursor peptides TrnA and TrnB (in the seminal work by Ross and Hill *et al.*, both the precursor peptides and the mature products were termed Trnα and Trnβ.^9^ Here the precursor peptides were named TrnA and TrnB to differentiate from mature sactipeptide products). Despite its early discovery, the biosynthetic pathway of thuricin CD remains unclear. It has generally been believed that TrnC and TrnD are each responsible for introducing three sactionine residues on a certain precursor peptide (*i.e.* TrnA or TrnB), in a way similar to the biosynthesis of two-component lanthipeptides (*e.g.* haloduracin).^5^ In this study, we demonstrate that, contrary to the commonly believed proposal, TrnC and TrnD employ a synergistic mechanism to coordinate the formation of thioether crosslinks on both TrnA and TrnB.

## Results and discussion

### *In vivo* studies in *E. coli*

**Fig. 2:**
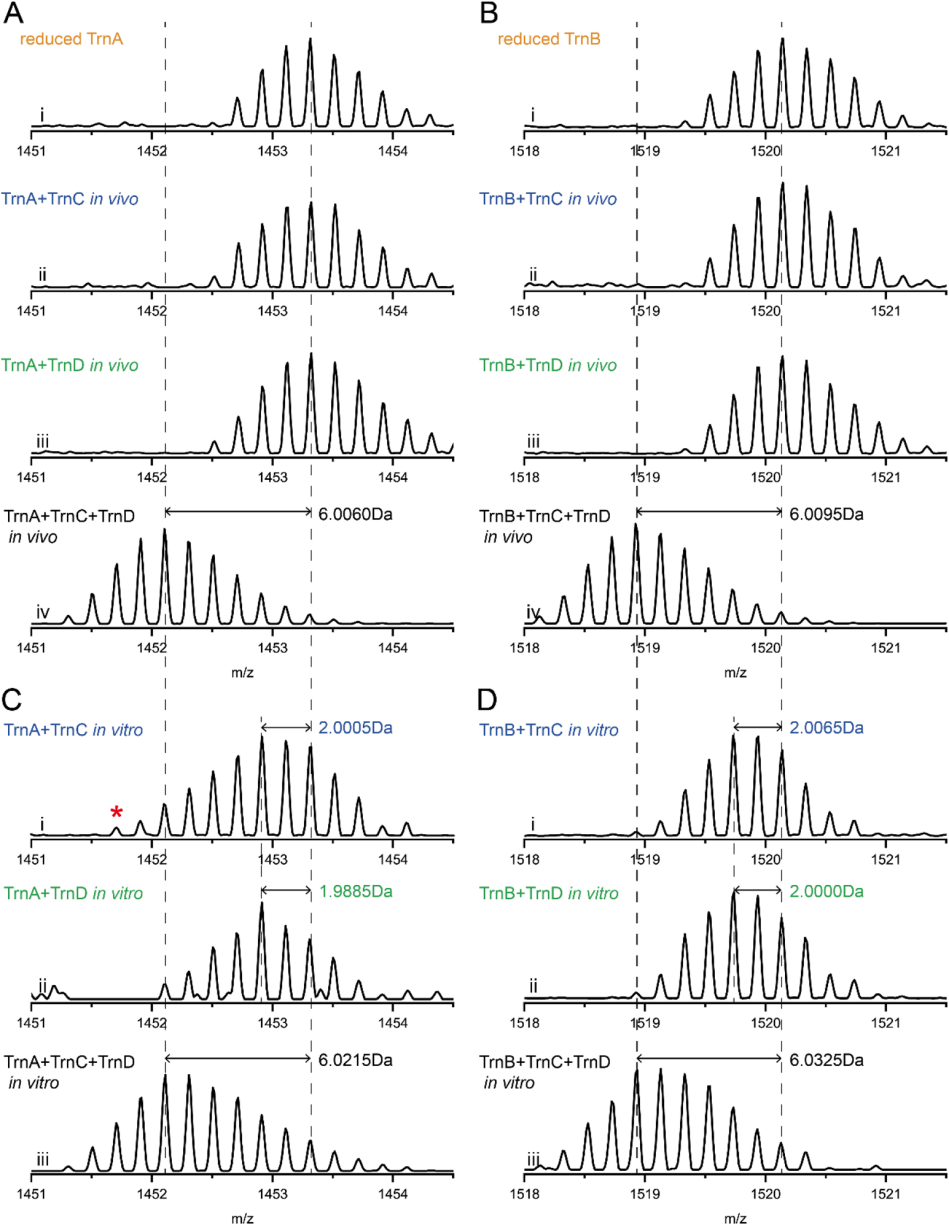
Synergistic activity of TrnC and TrnD. (A) MS spectra of TrnA obtained *in vivo* after TCEP reduction. (B) MS spectra of TrnB obtained similarly to (A). (C) MS spectra of TrnA modified in the *in vitro* analysis. The red asterisk indicates a small amount of the −4 Da product (*i.e.* t1-TrnA) produced by TrnC. See Fig. S14† for LC-HRMS and HR-MS/MS characterization of this product. (D) MS spectra of TrnB modified in the *in vitro* analysis.

We first set out to produce the precursor peptides in *E. coli*, a necessary step for the subsequent *in vivo* and *in vitro* study. Initially, TrnA and TrnB were expressed with an N-terminal 6× His tag, but the expected precursor peptides were not obtained, similar to prior attempts to express other RiPP precursor peptides.^37–40^ The peptides were then expressed as a fused protein with an N-terminal trigger factor (TF). After purification with Ni-NTA, the TF factor can be proteolytically removed by recombinant human rhinovirus HRV-3C protease, and the resulting peptides were obtained by precipitating the protein fraction using either heat denaturation or methanol treatment. The as-isolated TrnA and TrnB were −2 Dalton (Da) less than the expected molecular weight (Fig. S1 and S2†), suggesting the presence of a disulfide bond. Upon treatment with tris(2-carboxyethyl)phosphine (TCEP), the −2 Da products were reduced to unmodified peptides with the expected molecular weights (Fig. 2A and B, trace i), which can be fully derivatized using *N*-ethylmaleimide (NEM) (Fig. S1 and S2†), demonstrating the successful production of TrnA and TrnB in *E. coli*.

To investigate the role of TrnC and TrnD in thuricin CD biosynthesis, we coexpressed TrnA individually with TrnC or TrnD. HR-LCMS analysis of the TCEP-reduced products showed no modification occurred on TrnA (Fig. 2A, trace ii and iii), suggesting that TrnC and TrnD are unlikely to install a full set of thioether crosslinks on TrnA. Intriguingly, when TrnA was coexpressed with both TrnC and TrnD, the resulting peptide exhibited an apparent −6 Da modification (Fig. 2A, trace iv), which corresponds to formation of three thioether crosslinks on TrnA (hereafter this product was termed t3-TrnA, where t3 indicates 3 thioether bonds; similarly, t2 and t1 are used to denote the products with 2 and 1 thioether bonds, respectively). The t3-TrnA product was then treated with the endoprotease GluC to remove most of the leader peptide, giving a peptide trnα′ that is similar to trnα but containing two extra amino acids (*i.e.* IG) in the N-terminus (Fig. 1B). Although MS/MS analysis of peptides with multiple nested cycles is challenging, sactionine can partially cleave into a free Cys and an imine during MS/MS.^9^ LC-HRMS analysis of this peptide (trnα′) revealed the expected series of −6 Da, −4 Da and −2 Da y ions, which are consistent with the expected thioether crosslinks in trnα (Fig. S3†).

We also performed a similar set of coexpression studies with TrnB, and the result showed that neither TrnC nor TrnD alone was able to produce thioether crosslinks on TrnB (Fig. 2B, trace ii and iii). The expected −6 Da product was observed when TrnB was coexpressed with both TrnC and TrnD (Fig. 2B, trace iv). HR-MS/MS analysis of the GluC-treated product (trnβ′, which contains two extra amino acids VG in the N-terminus) showed that the three thioether crosslinks are consistent with those in trnβ (Fig. S4†). Together, our analyses demonstrate that TrnC and TrnD adopt a collaborative approach in the biosynthesis of the two-component sactibiotic thuricin CD, which together install the thioether crosslinks on the two precursor peptides TrnA and TrnB.

To test whether the thuricin CD variant obtained in *E. coli* was bioactive, we performed disk diffusion susceptibility tests with trnα′ and trnβ′ using *Bacillus cereus* as a test strain. The result showed apparent inhibition when trnα′ and trnβ′ were combined together, suggesting that the two peptides are bioactive. As expected, no activity was observed for trnα′ or trnβ′ alone (Fig. S5†). Careful quantification of the activity revealed that trnα′ and trnβ′ together exhibited a minimal inhibition concentration (MIC) of 3.1 μM against *B. cereus*, whereas no inhibition was observed for trnα′ or trnβ′ alone at 50 μM. These results demonstrate successful production of a thuricin CD variant in *E. coli*, suggesting that the presence of two extra N-terminal amino acids (*i.e.* IG in trnα′ and VG in trnβ′) likely does not have a direct impact on the antibiotic activity of thuricin CD (*i.e.* trnα and trnβ).^9^

### *In vitro* investigation

We next investigated the *in vitro* activity of TrnC and TrnD. Both proteins were overexpressed in *E. coli* with an N-terminal 6× His Tag, purified to near homogeneity and reconstituted under anaerobic conditions (Fig. S6†). Quantification analysis showed that TrnC contains 11.3 ± 0.2 mol Fe and 10.5 ± 0.5 mol S, whereas TrnD contains 9.4 ± 0.2 mol Fe and 8.2 ± 0.4 mol S. Further quantification by inductively coupled plasma atom emission spectroscopy (ICP-AES) analysis revealed 11.6 ± 0.2 mol and 9.1 ± 0.4 mol Fe for TrnC and TrnD, respectively. These results suggest that TrnC and TrnD likely contain three and two [4Fe–4S] clusters, respectively. To interrogate the rSAM chemistry of TrnC and TrnD, we incubated TrnC and TrnD respectively with SAM and sodium dithionite, and the reaction mixture was analyzed by LC-HRMS. The result revealed apparent production of deoxyadenosine (dAdoH) in both reactions with TrnC and TrnD (Fig. S7†), suggesting that TrnC and TrnD are both active rSAM enzymes. However, the SAM cleavage activity of TrnD is likely an evolutionary trait of the radical SAM superfamily and appears to be not relevant to thuricin CD biosynthesis (*vide infra*).

We next performed the assay by incubation of TrnA separately with TrnC and TrnD, in the presence of SAM and DTH (sodium dithionite). HR-LCMS analysis of the reaction mixture revealed that, as expected, the −6 Da product (corresponding to three thioether linkages) was not observed. Instead, TrnA in both reactions was mainly converted to the −2 Da product (Fig. 2C, traces i and ii). HR-MS/MS analysis showed that the −2 Da product does not contain a thioether crosslink; instead, it is an oxidized product containing a disulfide bond (Fig. S8 and S9†). Similar observations were also noted in parallel assays when the reaction was performed with TrnB (Fig. 2C, S10 and S11†). When TrnA was incubated with both TrnC and TrnD in the presence of other required components (*i.e.* SAM and dithionite), we observed the nearly complete conversion of TrnA to the fully modified −6 Da product (Fig. 2C, trace iii). Similarly, we found that TrnB can be fully modified to the −6 Da product in the presence of both TrnC and TrnD (Fig. 2D, trace iii). HR-MS/MS analysis revealed that the thioether crosslinks in the resulting −6 Da products of TrnA and TrnB are consistent with those in Trnα and Trnβ. (Fig. S12 and 13†). These analyses are consistent with the coexpression study discussed above, demonstrating that TrnC and TrnD work in a synergistic manner that together catalyze thuricin CD biosynthesis. Because each thioether crosslinking in sactipeptide biosynthesis requires consumption of a SAM,^41^ the turnover number of the *in vitro* reaction of TrnC and TrnD in this analysis is estimated to be ∼15 mol thioether crosslinks per mol enzyme.

In contrast to the coexpression study, a small amount of −4 Da product was observed in the reaction of TrnA with TrnC (Fig. 2C, trace i). Upon NEM treatment and GluC digestion, HR-MS/MS analysis indicated that this product contains a thioether crosslink between C13 and S21 (Fig. S14†), suggesting that TrnC alone can install a thioether crosslink on TrnA. This observation is reminiscent of our previous study in the study of thuricin Z.^25^ Similar to thuricin CD, the biosynthetic gene cluster of thuricin Z also encodes two rSAM enzymes ThzC and ThzD.^25,26^ Although the *in vitro* activity of the two enzymes is lower, fully modified ThzA (thuricin Z precursor) can be observed with the increased enyzme concentration of TnzC.^25^ We hence conducted the TrnA reaction with an increased TrnC concentration of 100 μM (5-fold higher than that in typical assays) and performed the reaction with prolonged incubation time. LC-HRMS analysis of the GluC-digested product clearly revealed the production of fully modified 3t-TrnA (Fig. S15†). However, no thioether crosslinking of TrnA was observed with the increased concentration of TrnD. These findings indicate that although TrnC and TrnD function synergistically, TrnC alone is capable of generating the fully modified product, albeit with markedly reduced efficiency (<5% relative to the TrnC–TrnD combination).

**Fig. 3:**
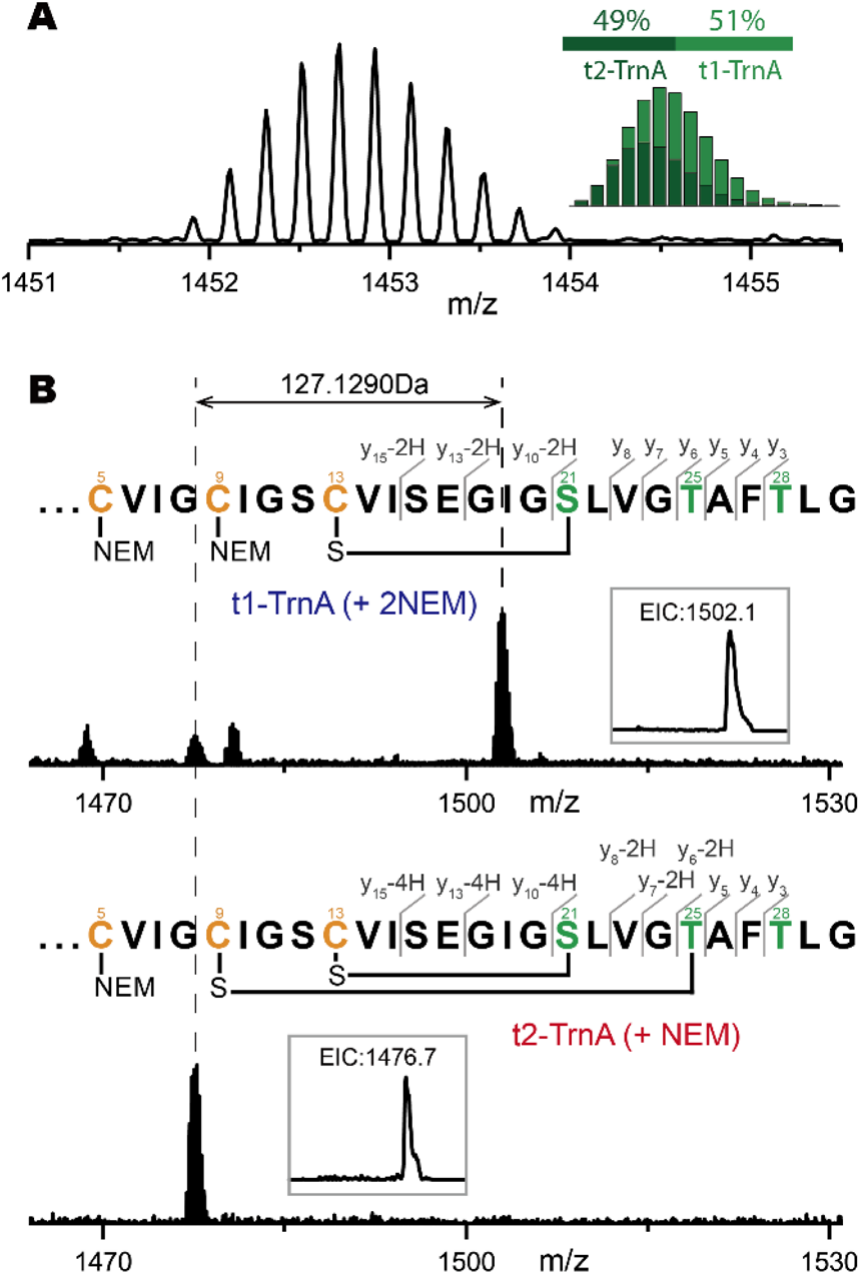
The reaction directionality in TrnA modification. (A) HR-MS spectrum of TrnA modified with TrnC and TrnD *in vitro*. The inset shows an approximately 1 : 1 ratio between the −2 Da product (t1-TrnA) and the −4 Da product (t2-TrnA) (calculated based on isotopic distribution). The reaction assay was performed by incubation of 100 μM TrnA with 20 μM TrnC, 20 μM TrnD, 2 mM DTH, 4 mM DTT and 1 mM SAM. Parallel reactions were incubated at 30 °C for 1 h (top) or 3 h (bottom) and then terminated with an equal volume of methanol. The mixture was treated with TCEP and analyzed by LC-HRMS after removal of protein precipitate by centrifugation. A time course analysis for TrnA modification is also shown in Fig. S16.† (B) LC-HRMS and HR-MS/MS analysis of the NEM-derivatized product obtained in the 1 h reaction. The insets show the extracted ion chromatograms (EICs) of the corresponding NEM-derivatized products.

To investigate the directionality in the formation of thioether crosslinks, we performed time course analysis of the TrnA reaction (Fig. S16†). When the 1-hour reaction was terminated by methanol precipitation and treated with TCEP to reduce any disulfide bonds, LC-HRMS analysis revealed a mixture of −2 Da and −4 Da products (corresponding to t1-TrnA and t2-TrnA) in approximately a 1 : 1 ratio (Fig. 3A). The reaction product was then treated with NEM and analyzed by LC-HRMS and HR-MS/MS (Fig. 3B), and the result showed that the thioether crosslink in t1-TrnA is formed between C13 and Ser21 (Fig. S17†). This observation is consistent with the product observed in the assay with TrnC alone (Fig. S14†), suggesting that the thioether crosslink between C13 and S21 is formed first in Trnα biosynthesis. Consistently, t2-TrnA contains a second thioether crosslink between C9 and T25 (Fig. S18†). After prolonging the incubation to 3 hours, all t1- and t2- intermediates were fully converted to the fully modified t3-TrnA (Fig. 3A). These results suggest that the thioether crosslink formation proceeds through the sequential processing of Cys residues in a C-to-N manner (*i.e.* C13 → C9 → C5 in TrnA).

### TrnC and TrnD form a stable protein complex

The coordinated action of TrnC and TrnD implies the formation of a closely associated protein complex. We made a pRSFduet-1-derived construct that expresses a 6× His-tagged TrnC and a non-tagged TrnD. Following expression in *E. coli*, the resulting cell lysate was subjected to Ni-NTA purification, and subsequent SDS-PAGE analysis clearly revealed the presence of both TrnC and TrnD with an approximate 1 : 1 stoichiometry in the purified fraction (Fig. S19†). We also combined the cell lysates expressing the His-Tagged TrnD with that of expressed non-Tagged TrnC. Subsequent SDS-PAGE analysis of the Ni-NTA purified product revealed the copurification of both TrnC and TrnD (Fig. S19†). These findings strongly support the formation of a robust protein complex between the two proteins.

**Fig. 4:**
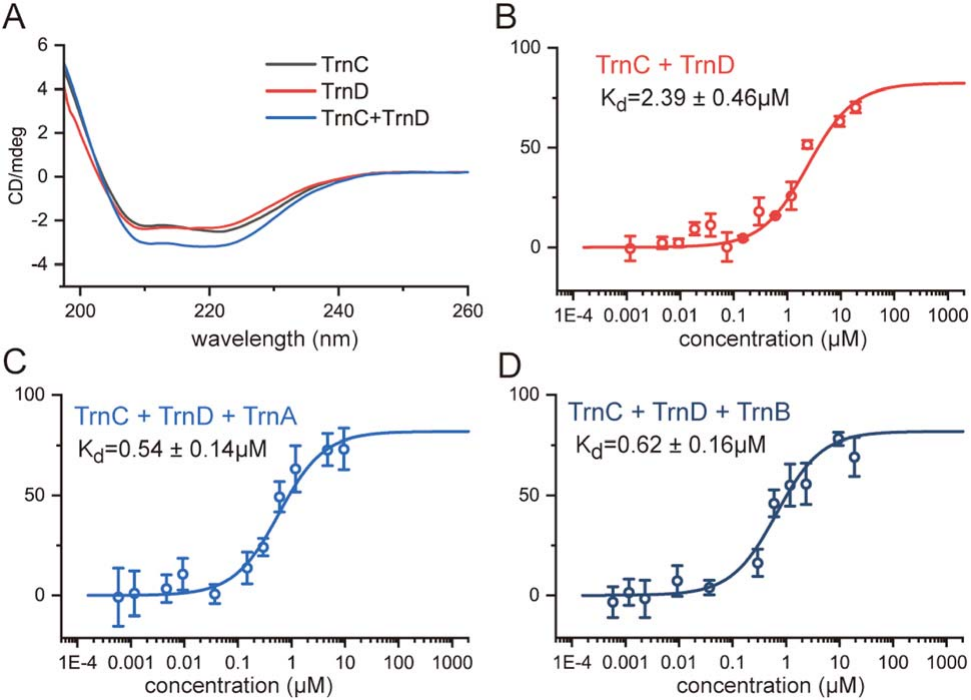
TrnC and TrnD form a stable protein complex. Formation of a robust protein complex between TrnC and TrnD in thuricin CD biosynthesis, showing (A) CD spectra of TrnC, TrnD, and a 1 : 1 mixture of TrnC and TrnD, and MST analyses of the fluorescently labeled TrnC with (B) TrnD, (C) TrnD and TrnA, and (D) TrnD and TrnB.

We next performed circular dichroism (CD) spectroscopy analysis of TrnC and TrnD. The results indicated that TrnC and TrnD exhibited similar CD spectra individually (Fig. 4A). However, when mixed, the CD signal showed a significant increase (Fig. 4A), strongly suggesting that TrnC and TrnD form a complex to result in structural and/or conformational changes. This observation supports that TrnC and TrnD form a robust protein complex.

To quantitatively evaluate the interaction between TrnC and TrnD, we performed microscale thermophoresis (MST) experiments to elucidate the dissociation constant (*K*_d_) between the two proteins. To this end, TrnC was fluorescently labeled with RED-NHS, and a series of diluted TrnD solutions were mixed with TrnC within capillaries for MST analysis. This analysis revealed a *K*_d_ of 2.39 ± 0.46 μM between TrnC and TrnD (Fig. 4A). Further analysis using an equimolar mixture of TrnD and TrnA revealed a 4-fold reduction in *K*_d_ to 0.54 ± 0.14 μM. Similarly, the analysis with an equimolar mixture of TrnD and TrnB revealed a *K*_d_ of 0.62 ± 0.16 μM (Fig. 4A). These findings demonstrate that TrnC and TrnD form a stable protein complex, and the presence of the precursor peptide (*i.e.* TrnA or TrnB) significantly strengthens the interaction, leading to the formation of a tighter ternary complex. We also performed MST analysis to assess the interactions between TrnA and TrnB with TrnC and TrnD individually. This analysis revealed *K*_d_ values between 2.1 and 5.4 μM, suggesting that TrnA and TrnB also have notable interactions with both TrnC and TrnD independently (Fig. S20†). We noted that prolonged air exposure of the sample led to an apparent increase in the *K*_d_ values, which is likely due to the degradation of the air sensitive Fe–S clusters in the proteins. Given that the MST analysis was performed under aerobic conditions, it is anticipated that the actual *K*_d_ values may be lower than those reported here.

### The interaction within the protein complex of TrnC and TrnD

**Fig. 5:**
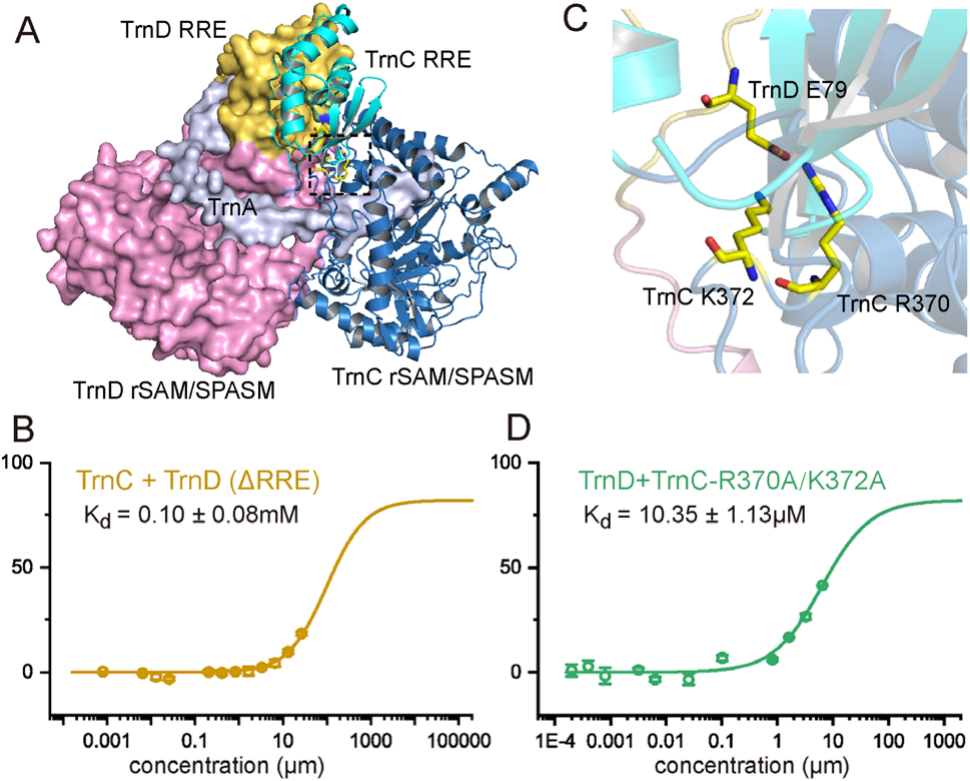
The interaction between TrnC, TrnD, and their substrate TrnA. (A) The AlphaFold 3 structure of the ternary structures of TrnC, TrnD, and TrnA. The RRE motif of TrnC and TrnD are shown in cyan and yellow, and the rSAM/SPASM domain of TrnC and TrnD are shown in blue and magenta. TrnA is shown in gray. (B) MST analysis of the fluorescently labeled TrnC with the RRE-deletion mutant of TrnD (TrnD(ΔRRE)). (C) Zoom-in on the boxed region in panel (A), showing the interaction between TrnD E69 and TrnC R370 and K372. (D) MST analysis of the fluorescently labeled TrnD with the R370A/K372A mutant of TrnC.

To investigate the detailed interaction within the protein complex, we performed AlphaFold 3 analysis to predict the TrnC–TrnD binary structure and TrnC–TrnD–TrnA ternary structure.^42^ The overall ternary structure is highly similar to that of the binary structure (Fig. S21†), with TrnA occupying the cavity between the rSAM/SPASM domains of TrnC and TrnD (Fig. 5A). TrnC and TrnD interact with each other primarily through the interaction between the two RiPP precursor peptide recognition element (RRE) domains^43^ of the respective proteins (Fig. 5A). TrnC appears to have a canonical RRE domain, featuring an N-terminal three β-strands and a following winged helix–turn–helix (wHTH) structure.^44,45^ In contrast, the predicted RRE domain of TrnD does not have a wHTH structure and is different from those of the canonical RREs (Fig. 5A and S21†).

**Fig. 6:**
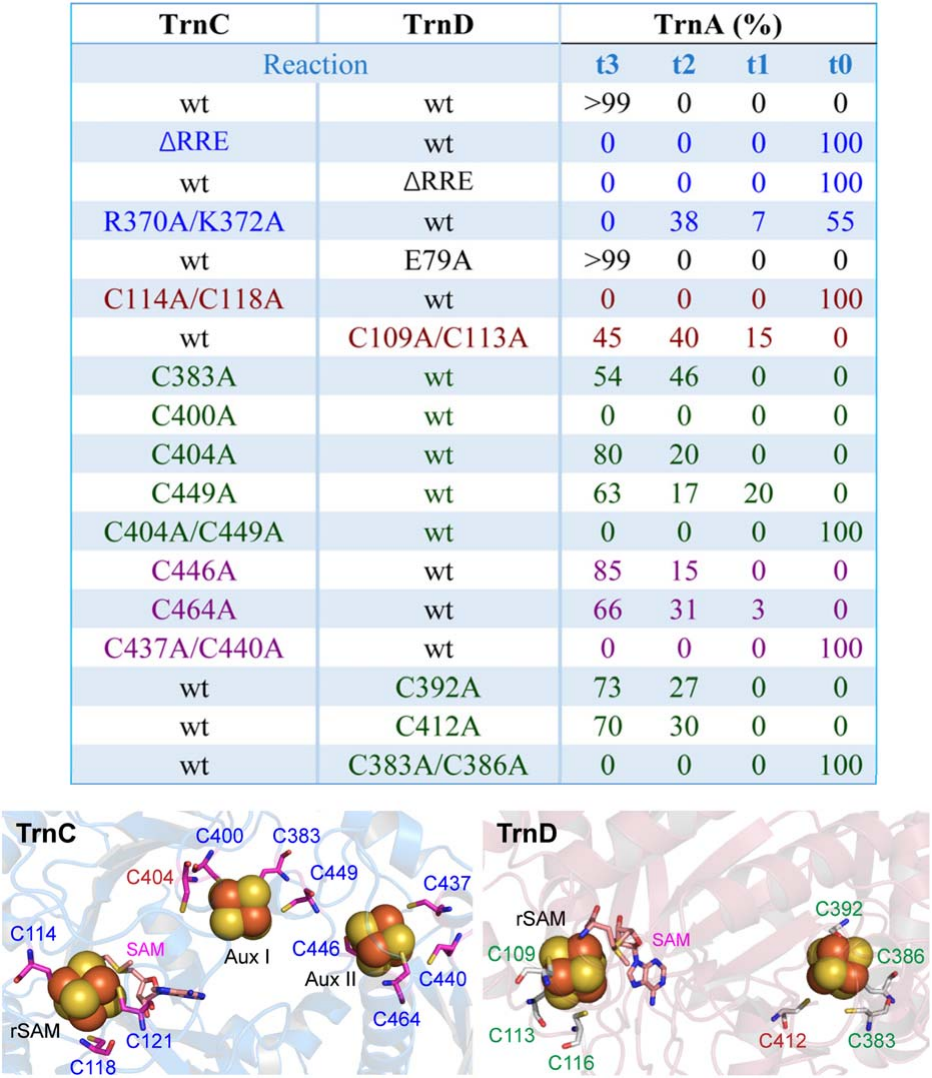
A summary of the *in vitro* studies performed for TrnC and TrnD mutants. For a clear illustration of the mutants in relation to Fe–S binding, the predicted [4Fe–4S] clusters in TrnC and TrnD are shown below. The mutants in relation to protein interactions are shown in blue. The mutants with the impaired rSAM cluster, Aux I, and Aux II are shown in brown, green, and purple, respectively. The details for sample preparation and analysis of different TrnA species are discussed in Fig. S22,† and the results are shown in Fig. S23–S25.† Since TrnA and TrnB exhibited similar activity in the reactions, we primarily focused on TrnA to assess the activity of various mutants. The activity of each mutant was also evaluated in coexpression studies, yielding comparable results (Fig. S26†). The binding of the [4Fe–4S] clusters was predicted through structural alignment of the AlphaFold 3 structure with the crystal structure of ranthipeptide biosynthetic enzyme CteB. The C404 in TrnC, which is not conserved in TrnC-like enzymes, is highlighted in brown.

To validate the critical role of the RRE motif in enzyme interaction, we removed the RRE motif from both TrnC and TrnD, generating the TrnC(ΔRRE) and TrnD(ΔRRE) mutants. Both mutant proteins were expressed in *E. coli* and purified to near homogeneity (Fig. S6†), indicating that the removal of the RRE motif does not affect protein solubility. However, subsequent MST analysis revealed that the *K*_d_ values for TrnC and TrnD(ΔRRE) increased to 0.1 ± 0.08 mM, which is more than 40-fold compared to the wild type TrnCD (Fig. 5B), and the *K*_d_ value for TrnC(ΔRRE) and TrnD was too high to be measured. Consistent with these findings, biochemical assays showed that no thioether crosslinking was formed by the RRE-deletion mutants (Fig. 6 and S22–S25†).

Among the interactions between TrnC and TrnD, a notable interaction occurs between E79 of TrnD and R370/K372 of TrnC (Fig. 5C). To test the importance of this interaction, we generated a TrnC R370A/K372A mutant. Pull-down assays showed a significant decrease in the interaction between TrnD and this TrnC mutant (Fig. S27†). MST analysis further supports this result, revealing a *K*_d_ of 10.25 ± 0.74 μM that is 5-fold higher than that of the wild type TrnC–TrnD complex (Fig. 5D). Consistent with this, only t1-TrnA and t2-TrnA were detected in the assay with TrnD and the TrnC R370A/K372A mutant (Fig. S24 and S25†), and no t3-TrnA was observed even after prolonged reaction times (Table S1 and Fig. S23†). Interestingly, when testing the E79A mutant of TrnD with wild type TrnC, we observed a slight increase in their interaction (Fig. S20†), indicating a complex and dynamic interaction between the two enzymes.

### Distinct roles of TrnC and TrnD rSAM chemistry in thuricin CD biosynthesis

The rSAM/SPASM enzymes contain a radical SAM domain, characterized by a partial (β/α)_6_ triose-phosphate isomerase (TIM) barrel, and a C-terminal SPASM domain that houses one or more auxiliary [4Fe–4S] clusters. Sactisynthases, the enzymes responsible for producing sactionine residues, use the rSAM [4Fe–4S] ([4Fe–4S]_rSAM_) to cleave SAM to generate a 5′-deoxyadenosyl (dAdo) radical.^46^ This radical then abstracts the α hydrogen atom from the targeted to-be-crosslinked residue,^47,48^ which is typically located in the C-terminal region of the core peptide. The resulting Cα radical then engages in the formation of a thioether link with a Cys residue located in the N-terminal region of the core peptide. The precise mechanism underlying sactionine formation remains unclear with different hypotheses proposed.^41,47–50^

The coordinated action of TrnC and TrnD in thuricin CD biosynthesis presents a highly unique example of enzyme synergy. To the best of our knowledge, the synergistic action of two functionally identical enzymes (in this case, sactisynthases), accomplishing a task that generally can be done by a single enzyme, is unprecedented in RiPP biosynthesis. In the AlphaFold 3 model, the core peptide, especially the crosslinking residues, predominantly interacts with TrnC, while TrnD primarily binds to the leader peptide (Fig. 5A). This suggests that the rSAM activity of TrnC plays a more critical role than that of TrnD in the formation of sactionine during thuricin CD biosynthesis.

To test this hypothesis, we disrupted [4Fe–4S]_rSAM_ by replacing the two Cys residues in the CxxxCxxC motif of TrnC and TrnD with Ala, generating the TrnC (C114A/C118A) and TrnD (C109A/C113A) mutants. Both mutant enzymes were expressed in *E. coli* and purified to near homogeneity, indicating that disrupting the rSAM cluster apparently does not affect protein solubility (Fig. S6†). Biochemical assays were then performed with TrnA in the presence of TrnC (C114A/C118A) and TrnD, as well as TrnC and TrnD (C109A/C113A). The results showed that sactionine formation was completely abolished in the TrnC (C114A/C118A) mutant (Fig. S23–S25†), highlighting the essential role of the [4Fe–4S]_SAM_ cluster in TrnC for thuricin CD biosynthesis. Further CD spectroscopy analysis showed that TrnC (C114A/C118A) has a highly similar spectrum to that of wild type enzyme (Fig. S28†), indicating the conformational integrity of the mutant protein. Interestingly, the TrnD (C109A/C113A) mutant still produced fully modified t3-TrnA with TrnC, though with slightly reduced activity (Fig. 6 and S23†). These findings, consistent with the AlphaFold 3 model (Fig. 5A), suggest that while both TrnC and TrnD can cleave SAM and produce dAdoH, the rSAM chemistry of TrnC is primarily responsible for thioether crosslinking in thuricin CD biosynthesis, whereas the rSAM chemistry of TrnD may not be directly involved in this process.

### Mutagenesis of Cys residues required for binding of auxiliary Fe–S clusters

The SPASM domain of TrnC contains a conserved seven-Cys motif C^383^x_11_Gx_4_Cx_36_Cx_2_Cx_5_Cx_2_Cx_14_C^464^ (Table S1†), corresponding to the Cx_9−15_Gx_4_Cx_*n*_Cx_2_Cx_5_Cx_3_Cx_*n*_C motif known to coordinate two auxiliary [4Fe–4S] clusters (Aux I and II).^51,52^ In contrast, TrnD has a truncated SPASM domain (TWITCH) with a three-Cys C^383^x_2_Cx_5_C^392^ motif likely for binding a single auxiliary [4Fe–4S] cluster. Genome mining using RODEO^53^ identified 9 biosynthetic gene clusters likely encoding sactipeptides similar to thuricin CD (Fig. S29†). Multiple sequence alignments showed that the seven-Cys motif is conserved in all TrnC-like enzymes (Fig. S30†), while the three-Cys motif and additional Cys (*e.g.* C412 in TrnD) are conserved in all the TrnD-like enyzmes (Fig. S31†). In the crystal structure of CetB and SuiB, two rSAM/SPASM enzymes in RiPP biosynthesis, Aux I binds to three Cys residues while Aux II binds four Cys residues (Fig. S32†).^45,54^ Structural alignment with the CteB crystal structure suggests that in TrnC, C383, C400, and C449 likely bind Aux I and C437, C440, C446, and C464 likely bind Aux II (Fig. 6). Interestingly, C404, which is not conserved in TrnC-like enzymes (Fig. S30†), is also positioned near Aux I. The residues C383, C386, and C392 likely bind the auxiliary [4Fe–4S] in TrnD, with C412 also in close proximity to this cluster (Fig. 6).

To investigate the role of the auxiliary [4Fe–4S] clusters in thuricin CD biosynthesis, we replaced the Cys residues involved in binding these clusters in TrnC and TrnD with Ala, respectively. All mutant proteins were expressed and purified to near homogeneity (Fig. S6†), indicating that disrupting the [4Fe–4S]_au_ cluster binding does not apparently affect protein solubility. The mutant enzymes were analyzed with the wild type partner protein (*i.e.* TrnD for TrnC mutants and TrnC for TrnD mutants). The result showed that, remarkably, while enzyme activity was abolished in some mutants, many retained significant activity. This contrasts with the general notion that auxiliary [4Fe–4S] clusters are essential for enzyme function.^51,52^ It is possible that nearby Cys residues (*e.g.* C404 in TrnC and C412 in TrnD) may partially compensate for cluster binding. Supporting this, all double mutants—where two Cys residues were simultaneously substituted with Ala—completely lost activity (Fig. 6). Owing to the complex binding patterns, multifaceted biochemical roles, and potential interconversion of [4Fe–4S] clusters (*e.g.*, [4Fe–4S] to [2Fe–2S]),^44^ further investigation is needed to fully understand their role in thuricin CD biosynthesis.

## Conclusions

In summary, we have characterized the role of two rSAM sactisynthases, TrnC and TrnD, in the biosynthesis of the two-component sactibiotic thuricin CD. Contrary to the conventional belief that each enzyme modifies a specific precursor peptide, our findings demonstrate their synergistic action within a tightly bound enzyme complex, which together catalyzes sactionine formation on both TrnA and TrnB. The fact that two functionally highly similar enzymes are acting synergistically to accomplish a task typically performed by a single enzyme is highly unique. One of the notable examples of such enzyme synergy is the tandem thioesterases involved in teixobactin biosynthesis.^55^ Additionally, we produced a thuricin CD variant in *E. coli* and found that the two amino acids in the N-termini of the thuricin CD peptides likely have minimal impact on antibiotic activity. Our study demonstrates the remarkably diverse pathways in sactipeptide biosynthesis and highlights the catalytic versatility of rSAM enzymes in producing RiPP natural products.

## Methods

High-performance liquid chromatography (HPLC) was conducted on a Thermo Scientific Dionex Ultimate 3000 system with a diode array detector. Liquid chromatography and high-resolution mass spectrometry (LC-HRMS) analysis was performed on a Q-ExactiveTM Focus Hybrid Quadrupole Orbitrap Mass Spectrometer (Thermo Fisher) equipped with a Dionex Ultimate 3000 HPLC system (Thermo Fisher). Nucleic acid and protein concentration determination was carried out on a micro-spectrophotometer (K5600) purchased from Kaiao Technology Development Co. Ltd (Beijing, China). Bacterial cell disruption was carried out by ultrasonication or by using a high-pressure homogenizer (FB-110X) purchased from Litu Ultra High-Pressure Equipment Co. Ltd (Shanghai, China). Anaerobic experiments were carried out in an anaerobic glove box (Coy Laboratory Product Inc., USA). The polymerase chain reaction was performed on a PCR thermocycler (ETC 811) purchased from Eastwin Scientific Equipments Co. Ltd (Suzhou, China). The microscale thermophoresis experiment was conducted on a Monolith instrument (NanoTemper Technologies). Iron content measurement was carried out on a ZA3000 atomic absorption spectrophotometer (Hitachi). All chemicals and biochemicals were purchased from commercial sources and used without further purification unless otherwise specified. For details of instrumental settings, procedures for data analysis, gene cloning, mutagenesis, protein expression and purification, *in vitro* biochemical assays, product purification, *in vitro* susceptibility test, protein–protein interaction assays, and genome mining analysis, please see the ESI.†

## Data availability

The data supporting this article have been included as part of the ESI.†

## Author contributions

Y. J., Y. H., X. L. and Q. Z. designed and performed the experiments. Q. Z. and Y. J. wrote the manuscript. All authors contributed to this work and have approved the final version of the manuscript.

## Conflicts of interest

There are no conflicts to declare.

## Supplementary Material

SC-OLF-D5SC01546D-s001
