# Supplementary material for: Synergistic action of two radical SAM enzymes in the biosynthesis of thuricin CD, a two-component sactibiotic
Source: Chem Sci. 2025 May 8;16(24):10722–30. doi: 10.1039/d5sc01546d (PMC12093266; doi:10.1039/d5sc01546d)
Supplement: SC-016-D5SC01546D-s001 [file SC-016-D5SC01546D-s001.pdf]

Supplementary Information (SI) for Chemical Science.
This journal is © The Royal Society of Chemistry 2025

Supplementary information

Synergistic action of two radical SAM enzymes in the biosynthesis of thuricin CD, a two-component sactibiotic

Yifei Jia[1,2], Yuanjun Han[1], XuXue Liu[2], and Qi Zhang[1,2]*

[1]Department of Chemistry, Fudan University, Shanghai, 200433

[2]National Engineering Research Center for Carbohydrate Synthesis, College of Chemistry and Materials, Jiangxi Normal University, Nanchang, Jiangxi, 330022

*Correspondence: qizhang@sioc.ac.cn

Supporting Information

Supporting Materials and Methods

Figures S1-S32

Tables S1-S2

Supporting Materials and Methods

Data analysis

LC-MS data was analyzed using Thermo Xcalibur v2.2 Qual Browser. Peptide MS and MS/MS, and peptide fragmentation by GluC digest are predicted using mMass (Version 5.5.0) (*1*). Experimental data are visualized using SigmaPlot 14.0 unless otherwise specified. All experimental data described in this study have been repeated successfully and at least two biological duplicates are conducted.

Chemicals, Biochemicals, and Bacterial strains

All chemicals and biochemicals were purchased from commercial sources and used without further purification unless otherwise specified. ACS/HPLC certified MeCN and MeOH were purchased from J&K Scientific (Beijing, China). Watsons water (distilled) was used as mobile phase solvent in the HPLC and LC-HRMS analysis. Kanamycin, ampicillin, chloramphenicol, isopropyl β-D-1-thiogalactopyranoside (IPTG), dithiothreitol (DTT), phenylmethylsulfonyl fluoride (PMSF), lysozyme and the culture media were from Sinopharm Chemical Reagent Co. Ltd (China) or Sangon Biotech Co. Ltd (Shanghai, China). Sodium dithioionate (DTH), $Fe(NH_4)_2(SO_4)_2 \cdot 6H_2O$ and Na_2S were from Adamas Reagent Co. Ltd (Shanghai, China). Tris(2-carboxyethyl) phosphine (TCEP) and phenylmethanesulfonyl fluoride (PMSF) were purchased from BBI life sciences corporation. N-ethylmaleimide (NEM) was purchased from Aladdin. Restriction enzymes (e.g. DpnI) were from New England Biolabs (Beijing, China). Endoproteinase Glu-C, recombinant human rhinovirus HRV-3C protease, and Bradford protein assay kit were purchased from Sangon Biotech Co. Ltd (Shanghai, China). Phanta super-fidelity DNA polymerase, DNA cleanup kit and ClonExpress II one step cloning kit were from CWbio Co. Ltd (Beijing, China) or Vazyme Biotech Co., Ltd (Nanjing, China). Ni-NTA resins were from Smart lifesciences (Changzhou, China, Ni Smart Beads 6FF: SA036100) or from GE Healthcare, USA. PD-10 minitrap G-25 columns were from GE Healthcare, USA. Gene synthesis was offered by Sangon

Biotech Co. Ltd (Shanghai, China). Primers were synthesized by Genewiz Co. Ltd (Suzhou, China) or Sangon Biotech Co. Ltd (Shanghai, China). *E. coli* DH5α cells and *E. coli* BL21(DE3) were purchased from Takara Biotechnology (Beijing, China) and TransGen Biotech (Beijing China).

Molecular biology

The gene sequences coding for TrnA, TrnB, TrnC, and TrnD were synthesized and cloned into the NdeI/XhoI restriction site of pET28a to afford pET28a-His$_6$-TrnA, pET28a-His$_6$-TrnB, pET28a-His$_6$-TrnC, and pET28a-His$_6$-TrnD. Since expression of TrnA and TrnB by using the pET28a-derivative vectors was unsuccessful, the trnα and trnβ genes were also inserted into the BamHI/HindIII site of pCold ™ TF vector to generate pCold-TrnA and pCold-TrnB, which express the precursor peptide as trigger factor (TF)-fused proteins. For coexpression analysis, *trnC* and *trnD* were inserted into the NdeI/XhoI restriction site in the multiple cloning site 2 (MCS2) of pRSFDuet-1 to generate pRSFDuet-TrnC and pRSFDuet-TrnD for expressing the non-tagged proteins. For coexpression both TrnC and TrnD, *trnC* was inserted into the NcoI/HindIII site within the multiple cloning site 1 (MCS1) of pRSFDuet-TrnD, generating pRSFDuet-TrnC-TrnD that produces both non-tagged TrnC and TrnD. For pull down analysis, *trnC* was inserted into the BamHI/HindIII site within the MCS1 of pRSFDuet-TrnD to generate pRSFDuet-His$_6$-TrnC-TrnD, which expresses the His-Tagged TrnC and non-Tagged TrnD.

All site-directed mutagenesis was performed using Phanta super-fidelity DNA polymerase and the resulting PCR reaction mixture was processed with DpnI to remove templates before transforming to chemically competent *E. coli* DH5α cells. The correct recombinant plasmids of all cloning steps were verified by sequencing technique offered by Genewiz Co. Ltd. or Sangon Biotech Co. Ltd. All primer sequences can be found in Table S1.

(Co)expression of Trnα or Trnβ in *E. coli*

E. coli BL21(DE3) cells were transformed with the precursor peptide-expression

plasmid (i.e. pCold-TrnA or pCold-TrnB) alone, or together with the sactisynthase-expression plasmid (i.e. pRSFDuet-TrnC, pRSFDuet-TrnD, or pRSFDuet-TrnC-TrnC) for coexpression. The resulting cell were grown for 10-15 h on LB agar plate (5 g/L yeast extract, 10 g/L tryptone, 10 g/L NaCl and 20 g/L agar) containing 100 μg/ml ampicillin (and 50 μg/ml kanamycin for coexpression) at 37 °C. Single colony was used to inoculate 10 mL of LB culture (5 g/L yeast extract, 10 g/L tryptone and 10 g/L NaCl) containing 100 μg/ml ampicillin and grown at 37 °C for 14–16 h. This culture was used to inoculate 1 L of LB culture containing 100 μg/ml ampicillin (and 50 μg/ml kanamycin for coexpression), and was grown for about 3hr at 37 °C at 150 rpm until OD_{600} reached ~0.8. Protein expression was then induced with the addition of 0.5 mM IPTG. For the case of co-expression, 0.5 mM $Fe(NH_4)_2(SO_4)_2{\bullet}6H_2O$ was additionally added to the culture. Expression was allowed to proceed for another 16-18 h at 18 °C at 50 rpm. Cells were harvested by centrifugation at 4,500 × g for 15 min, and washed with Tris lysis buffer (40mM Tris-HCl, 200mM NaCl, 5mM imidazole, 10% glycerol, pH 8.0). The cells were subsequently subjected to protein purification, or were flash-frozen by liquid nitrogen and stored at -80 °C upon further use.

To purify the TF-fused precursors, the cells were re-suspended in Ni-NTA lysis buffer (40mM Tris-HCl, 200mM NaCl, 5mM imidazole, 10% glycerol, pH 8.0), supplemented with PMSF (15 mg per L of initial cell culture) and lysozyme (120 mg per L of initial cell culture) and were then lysed using an ultrahigh pressure microfluidic homogenizer. on ice. Insoluble debris was then removed by centrifugation at 13000 × g for 55 min at 4 °C. The supernatant containing desired protein was then applied to a lysis-buffer pre-equilibrated Ni-NTA resin (3 mL resin per L of initial cell culture). The column was washed with 2 column volumes (CV) of Ni-NTA lysis buffer followed by 2 CV of Ni-NTA wash buffer (40 mM Tris-HCl, 200 mM NaCl, 50 mM imidazole, 10% glycerol, pH 8.0). The desired proteins were then eluted with Ni-NTA elution buffer (40 mM Tris-HCl, 200 mM NaCl, 300 mM imidazole, 10% glycerol, pH 8.0) and exchanged with the peptide desalting buffer (10 mM Tris, 25mM NaCl, 2.5% glycerol pH 8.0) using a PD-10 column. HRV-3C protease stock solution (1000 U) was then added to

the elution fraction (1: 200 (v/v) ratio), and the resulting solution was then kept overnight for digestion at 4 ^{o}C. The resulting solution was heat-inactivated at 95 °C for 10 min followed by freezing at -80 °C for 1 h, and was subjected to centrifugation (13000 × g for 15 min at 4 °C) to remove any precipitates. The resulting solutions containing TrnA or TrnB were directly used in downstream analysis or frozen at -80 °C for subsequent use.

Production of trnα' and trnβ'

The modified TrnA and TrnB solutions were mixed with GluC (2 mg/ml stock solution) in a 50:1 (v/v) ratio and the proteolytic reaction was proceeded at room temperature for 2hr. The reaction mixture was subsequently quenched by methanol, and was lyophilized after centrifugation to remove any protein precipitate. The resulting sample was then dissolved in 30% isopropanol and injected onto a reverse phase Phenomenex Aeris PEPTIDE XB-C18 column (250 x 10 mm, 5 μm). A gradient elution operating at 3 mL/min was used with solvent A (distilled water containing 0.1% TFA) and solvent B (MeCN) under the following condition: t = 0 min, 10% B; t = 15 min, 45% B; t = 28 min, 90% B; t = 35 min, 90% B; t = 37 min, 10% B. The fractions containing desired peptide fraction were pooled, dried by rotary evaporation and lyophilized to dryness upon use.

In vitro susceptibility test

The HPLC purified trnα' and trnβ' were dissolved in 30% isopropanol (a.q.). Antibacterial activity test of trnα' and trnβ' was performed with *Bacillus cereus* as the test strain according to procedures similar to those reported previously (*2*). Briefly, the liquid Brain Heart Infusion (BHI) agar (3.2 % BHI, 1.5% agar) was cooled to 42 °C and seeded with 100 μL of dense overnight culture (approximately 10^8-10^9 CFU mL^{-1}) of *Bacillus cereus*. After agar solidification in a Petri dish, samples (5 μL 100 μM trnα', or 5 μL 100 μM trnβ', or 5 μL 100 μM trnα' and 5 μL 100 μM trnβ') were applied to a small sterilized filter paper, which was placed onto the surface of the medium, and the plates were incubated at 30 ^{o}C for 15-20 h. Kanamycin and 30% isopropanol were used

as a positive and negative controls, respectively. For minimal inhibition concentration (MIC) analysis, overnight culture of overnight culture (approximately 10^8-10^9 CFU mL^{-1}) of *Bacillus cereus* were diluted 1000-fold and transferred to a 96-well plate, each containing 80 μL LB culture. 16 uL 500 μM trnα' and 64 uL LB culture; 16 μL 500 μM trnβ' and 64 uL LB culture; and 16 uL 500 μM trnα', 16 μL 500 μM trnβ' and 48 uL LB culture, were each added to a well and mixed, respectively. Serial dilutions were performed by transferring 80 μL sample from one well to the next. Kanamycin and 30% isopropanol were also used as a positive and negative controls, respectively. The plates were incubated at 30 °C for 16 hours. The OD_{600} was read on a SP-Max 2300A plate reader. MICs were designated as the lowest concentration that produced an increase of less than 10% in OD over that of the adjacent kanamycin well.

Expression, purification and reconstitution of TrnC and TrnD

E. coli BL21(DE3) cells were co-transformed with the corresponding plasmid (i.e. pET28a-TrnC or pET28a-TrnD) with the pSUF plasmid, the latter encodes the Isc iron-sulfur assembly system to facilitate *in vivo* [4Fe-4S] cluster assembly (*3*). The overall procedure for TrnC or TrnD expression is similar to that described in "(Co)expression of Trnα or Trnβ in *E. coli*" section, for coexpression, with only variation in the antibiotics in the culture: here 50 μg/ml kanamycin and 25 μg/ml chloramphenicol were used. Cells were harvested by centrifugation at 4,500 × g for 15 min, washed with Tris lysis buffer (40mM Tris-HCl, 200mM NaCl, 5mM imidazole, 10% glycerol, pH 8.0), and were subsequently subjected to protein purification, or were flash-frozen by liquid nitrogen and stored at -80 °C upon further use.

All the purification and reconstitution steps were carried out anaerobically in a glove box with O_2 concentration lower than 5 ppm. All buffers used for here were degassed and stored for 24–48 h in anaerobic chamber before use. The cells were re-suspended in Ni-NTA lysis buffer (40mM Tris-HCl, 200mM NaCl, 5mM imidazole, 10% glycerol, pH 8.0), supplemented with PMSF (15 mg per L of initial cell culture) and lysozyme (120 mg per L of initial cell culture) and were then lysed by sonication (every 3 s sonication at 70% intensity followed by 9 s interval) for 60 min on ice. Insoluble

debris was then removed by centrifugation at 13000 × g for 55 min at 4 °C. The supernatant was then applied to a lysis-buffer pre-equilibrated Ni-NTA resin (3 mL resin per L of initial cell culture). The column was washed with 2 column volumes (CV) of Ni-NTA lysis buffer followed by 2 CV of Ni-NTA wash buffer (40 mM Tris-HCl, 200 mM NaCl, 50 mM imidazole 和 10% glycerol, pH 8.0). The desired His_6-tagged protein was then eluted with Ni-NTA elution buffer (40 mM Tris-HCl, 200 mM NaCl, 300 mM imidazole, 10% glycerol, pH 8.0). The elution was concentrated by a 30,000 Da molecular weight cut-off Amicon Ultra centrifugal filter. The concentrated protein was exchanged into the protein desalting buffer (10 mM Tris, 25 mM NaCl, 10% glycerol, pH 8.0) using a PD-10 desalting column per manufacturer's protocol. Protein concentration was quantified using Bradford assay kit based on bovine serum albumin (BSA) as a standard.

Reconstitution of the [4Fe-4S] clusters was also performed under strict anaerobic condition (O_2 < 5 ppm) according to a procedure similar to that reported previously.(*4*) Briefly, solutions of dithiothreitol (1 M), sodium sulfide ($Na_2S{\cdot}9H_2O$, 50 mM), and ferrous iron ($(NH_4)_2Fe(SO_4)_2$, 50 mM) were prepared freshly prior to reconstitution. DTT was added to protein solution to a final concentration of 10 mM and $(NH_4)_2Fe(SO_4)_2$ was carefully added to a final concentration of 1 mM. After a 10 min incubation on ice, the mixture was supplemented with $Na_2S{\cdot}9H_2O$ repeatedly for three times until a final concentration reaching 1 mM, each time incubated on ice for 20 min. The reconstitution reaction was further proceeded at 4 °C overnight, and the excess iron and sulfur were removed via PD-10 column as described above. After concentration by a 30,000 Da molecular weight cut-off Amicon Ultra centrifugal filter, the protein was used directly in the biochemical assays. Previously-described methods were used to determine the content of Fe and labile S.(*5, 6*) The Fe content of TrnC and TrnD was also quantified by inductively coupled plasma atom emission spectroscopy (ICP-AES) as previously described,(*7*) which gave similar results.

SAM cleavage assays with TrnC or TrnD

2 mM SAM, 4 mM DTH and ~10 µM TrnC or TrnD were mixed in 20 mM Tris-HCl buffer (pH 8.0). Reaction volumes were typically 50 µL and were maintained at 28 °C in glove box for 30 min prior to quenching. The reactions were quenched by formic acid (v/v 1:10). After removal of the protein precipitates by centrifugation, the supernatant was sent for the LC-HRMS analysis.

Peptide modification assays *in vitro*

Biochemical assays were performed under anaerobic condition (O_2 < 5 ppm) by incubation of 200 µM precursor peptide substrate (i.e. TrnA or TrnB) with 1 mM SAM, 2 mM DTH, 4 mM DTT, and ~20 µM modifying enzyme(s) (i.e. TrnC, or TrnD, or TrnC + TrnD). Reaction volumes were typically 50 µL and were maintained at room temperature in glove box for 3 hr prior to quenching by an equal volume of methanol. For investigation the reaction directionality, the same assay was performed with TrnA for 1 h. After removal of protein precipitate by centrifugation at 12000 x g for 10 min, the supernatants were directly analyzed by LC-HRMS. For investigation the reaction directionality, the same assay was performed with TrnA for 1 h. After removal of protein precipitate by centrifugation at 12000 x g for 10 min, the supernatants were directly analyzed by LC-HRMS.

AlphaFold-Multimer analysis

The heterodimeric structure of TrnC and TrnD in complex with precursor peptide Trnα or Trnβ was predicted by AlphaFold-Multimer(*8*) enabled in ColabFold(*9*), a publicly available Jupyter notebook that replaces the homology detection and MSA pairing of AlphaFold2 with MMseqs2. The resulting unrelaxed models of the structure complexes were analyzed by Pymol software package (*10*).

Analysis of the products in co-expression and mutagenesis studies.

Peptide coexpression and purification were performed according to those described in "(Co)expression of Trnα or Trnβ in *E. coli*" section. The TF-fused peptide eluted from Ni-NTA purification was treated with 5 mM NEM for 10min at room temperature, and

was subjected to desalting by PD-10 column using peptide desalting buffer (10 mM Tris, 25 mM NaCl, pH 8.0). The resulting peptide elution was concentrated through a 10,000 MWCO regenerated cellulose membrane. After overnight cleavage by HRV-3C at 4℃ for 18h, the solution was incubated at 95 ℃ for 10 min, and the protein precipitate was removed by centrifugation. After monitoring the corresponding peptides in LC/MS, the products were then treated with GluC (final concentration 0.04 mg/ml). The proteolytic fragments of the modified TrnA and TrnB were then examined by LC-HRMS analysis (see Figure S18-S39 for details).

Microscale thermophoresis experiment

The interaction between TrnC and TrnD was measured by a NanoTemper monolith instrument at 20% LED power and 40% IR-laser power. TrnC, TrnD were changed to a HEPES buffer (50mM HEPES, 150mM NaCl, 10% glycerol 0.05% Tween20, pH = 8.2) in anaerobic chamber by using a PD-10 column, whereas TrnA, and TrnB were changed into the same HEPES buffer by ultrafiltration using a 10,000 MWCO regenerated cellulose membrane. 90μL 10 μM TrnC protein was incubated with 10μL 300 μM RED-NHS fluorescent molecules obtained from Nanotemper Company at RT for 30min. Free dyes were then removed using a gel column. The absorption values of the fluorescently labeled TrnC at A_{205} and A_{650} were measured by a UV-VIS spectrophotometer, and the protein concentration was calculated according. 80 nM RED-NHS-TrnC within two glass capillaries was transferred out of the anaerobic glove box to Monolith apparatus for parallel pretest. 16 double diluted protein solutions of TrnD were then mixed with 160 nM TrnC and absorbed successively by 16 glass capillaries for binary affinity test. Similar assays were also performed for an equal molar of TrnD + TrnA and TrnD + TrnB for the analysis of the ternary complex.

LC-HRMS analysis for peptide modification

LC-HRMS analysis in this study was performed with a Dionex UltiMate^{TM} 3000 HPLC system (Thermo scientific) coupled to a Q-ExactiveTM Focus Hybrid Quadrupole Orbitrap Mass Spectrometer (Thermo Fisher). The assay was carried out as follows

unless otherwise specified. 5 μL sample was injected onto the LC–MS system equipped with a LC column (BioBasic-8, Thermo Scientific, 100 x 2.1 mm, 5 μm). The MS spectra were acquired in positive ion mode, and the LC system was operated at 0.3 mL/min with solvent A (0.1% FA in distilled water) and solvent B (MeCN) under the following condition: t = 0 min, 2% B; t = 2 min, 2% B; t = 5.5 min, 40% B; t = 7.5 min, 40% B; t = 8 min, 95% B; t = 9.5 min, 95% B; t = 10.5 min, 2% B. The MS/MS spectra were acquired with a stepped collision energy (CE): 15, 30, 45 eV.

Genome mining

Protein sequence of TrnC was used to perform position-specific iterative (PSI)-BLAST in NCBI non-redundent (nr) protein database to retrieve sequence homologs (as of May 2024). The resulting ~100 TrnC-like proteins were analyzed by RODEO (*11*) web tool and scored by the "sacti/ranthi" scoring module (*12*) to retrieve genomic context annotation. The gene loci encoding the ~100 TrnC-like proteins were manually inspected and the selected biosynthetic gene clusters of sactipeptides were aligned and visualized by the clinker (*13*) web tool. Further multiple sequence alignment (MSA) of polypeptide sequences was performed with locally installed MAFFT software, using G-INS-I strategy (*14*).

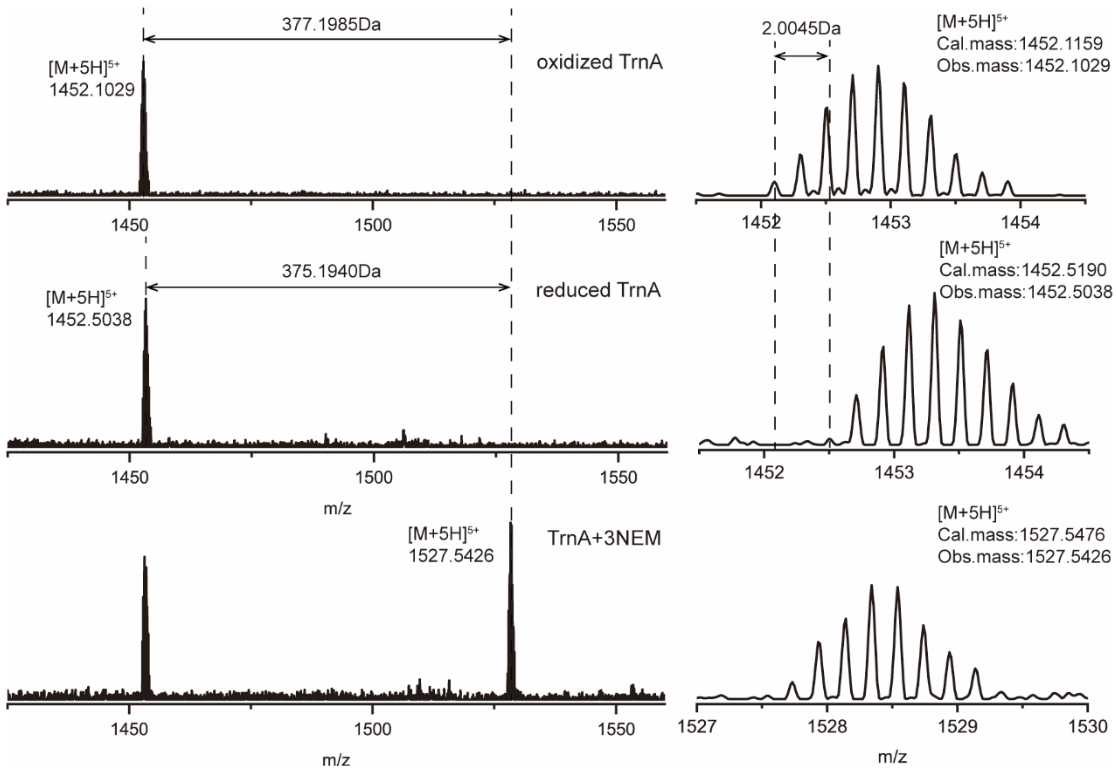

Figure S1. Mass spectra of TrnA produced in *E. coli*, showing the as-isolated TrnA (oxidized, with a disulfide bond), the TCEP-reduced TrnA, and the TCEP-reduced, NEM-derivatized TrnA.

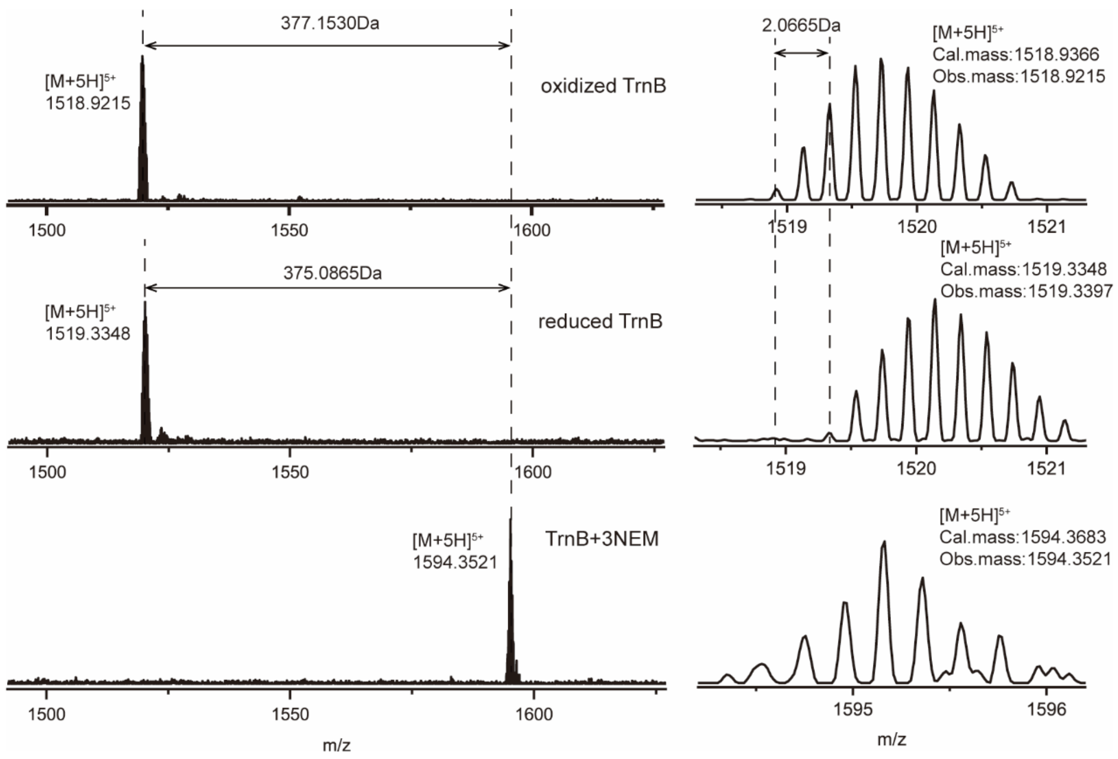

Figure S2. Mass spectra of TrnB produced in *E. coli*, showing the as-isolated TrnB (oxidized, with a disulfide bond), the TCEP-reduced TrnB, and the TCEP-reduced, NEM-derivatized TrnB.

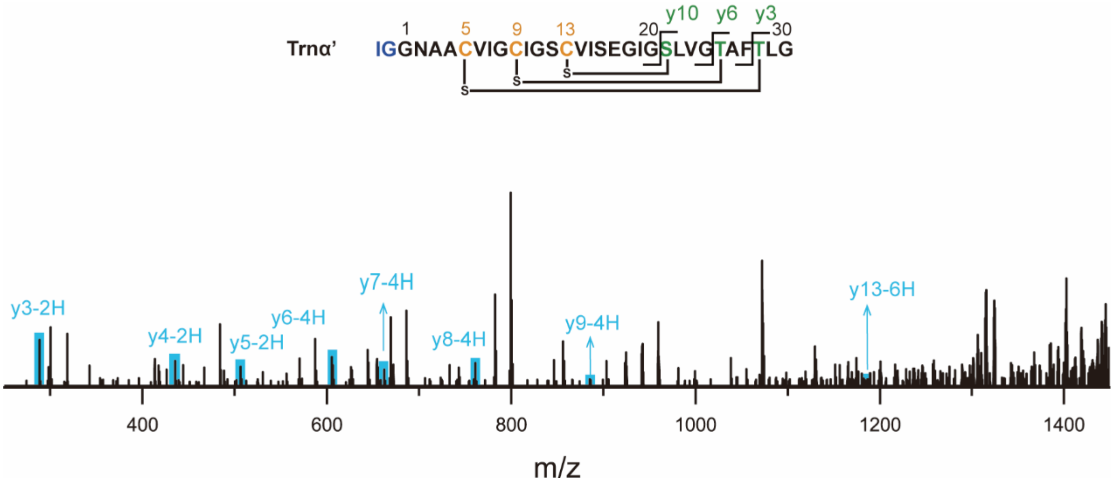

Figure S3. HR-MS/MS spectrum of Trnα', which contains two extra N-terminal amino acids (i.e. IG) compared to Trnα.

y ions	Sequence	Calculated Mass $[M+H]^+$	Observed Mass	Mass Difference (Da)	Error (ppm)
y3	**T**LG	290.1710	288.1568	2.0142	5.03
y4	F**T**LG	437.2395	435.2259	2.0136	4.71
y5	AF**T**LG	508.2766	506.2607	2.0159	0.49
y6	**T**AF**T**LG	609.3243	605.2896	4.0347	5.62
y7	G**T**AF**T**LG	666.3457	662.3112	4.0345	4.83
y8	VG**T**AF**T**LG	765.4141	761.3806	4.0335	2.89
y9	LVG**T**AF**T**LG	878.4982	874.4701	4.0281	3.66
y10	**S**LVG**T**AF**T**LG	965.5302	NA	NA	NA
y11	G**S**LVG**T**AF**T**LG	1022.5517	NA	NA	NA
y12	IG**S**LVG**T**AF**T**LG	1135.6358	NA	NA	NA
y13	GIG**S**LVG**T**AF**T**LG	1192.6572	1186.6057	6.0515	3.83
y14	EGIG**S**LVG**T**AF**T**LG	1321.6998	NA	NA	NA
y15	SEGIG**S**LVG**T**AF**T**LG	1408.7318	NA	NA	NA
y16	ISEGIG**S**LVG**T**AF**T**LG	1521.8159	NA	NA	NA

y ions of -2 Da, -4 Da, and -6 Da are highlighted by yellow, blue, and green, respectively.

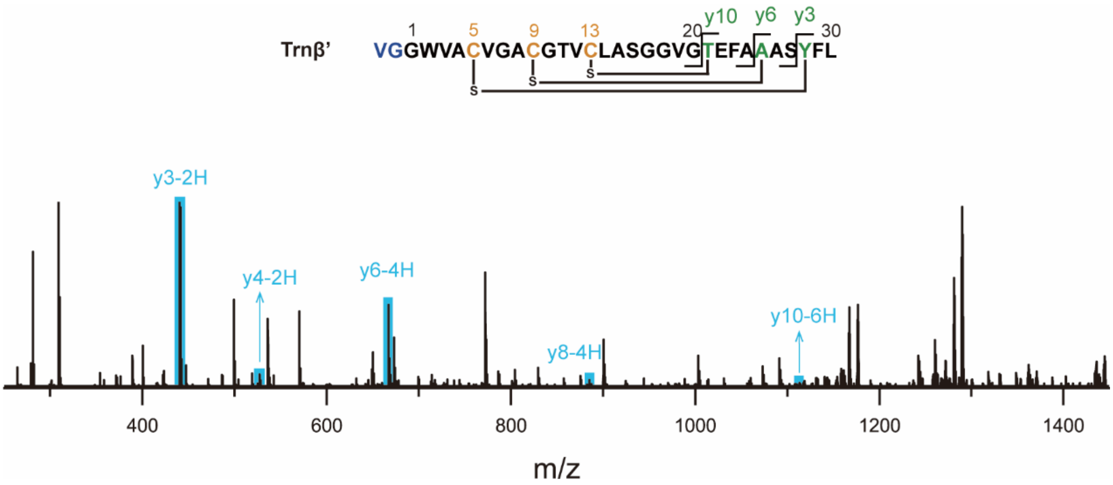

Figure S4. HR-MS/MS spectrum of Trnβ', which contains two extra N-terminal amino acids (i.e. VG) compared to Trnβ.

y ions	Sequence	Calculated Mass $[M+H]^+$	Observed Mass	Mass Difference (Da)	Error (ppm)
y3	<u>Y</u>FL	442.2336	440.2171	2.0165	1.93
y4	S<u>Y</u>FL	529.2657	527.2476	2.0181	4.65
y5	AS<u>Y</u>FL	600.3028	NA	NA	NA
y6	<u>A</u>AS<u>Y</u>FL	671.3399	667.3063	4.0336	3.45
y7	A<u>A</u>AS<u>Y</u>FL	742.3770	NA	NA	NA
y8	FA<u>A</u>AS<u>Y</u>FL	889.4454	885.4125	4.0329	1.81
y9	EFA<u>A</u>AS<u>Y</u>FL	1018.4880	NA	NA	NA
y10	<u>T</u>EFA<u>A</u>AS<u>Y</u>FL	1119.5357	1113.4833	6.0524	4.89
y11	G<u>T</u>EFA<u>A</u>AS<u>Y</u>FL	1176.5572	NA	NA	NA
y12	VG<u>T</u>EFA<u>A</u>AS<u>Y</u>FL	1275.6256	NA	NA	NA
y13	GVG<u>T</u>EFA<u>A</u>AS<u>Y</u>FL	1332.6470	NA	NA	NA
y14	GGVG<u>T</u>EFA<u>A</u>AS<u>Y</u>FL	1389.6685	NA	NA	NA
y15	SGGVG<u>T</u>EFA<u>A</u>AS<u>Y</u>FL	1476.7005	1470.6624	6.0381	6.02
y16	ASGGVG<u>T</u>EFA<u>A</u>AS<u>Y</u>FL	1547.7377	NA	NA	NA

y ions of -2 Da, -4 Da, and -6 Da are highlighted by yellow, blue, and green, respectively. Noted that the y11 ion is not consistent with the expected mass (-4 Da instead of -6 Da), which is likely derived from the parent iron 2t-TrnB.

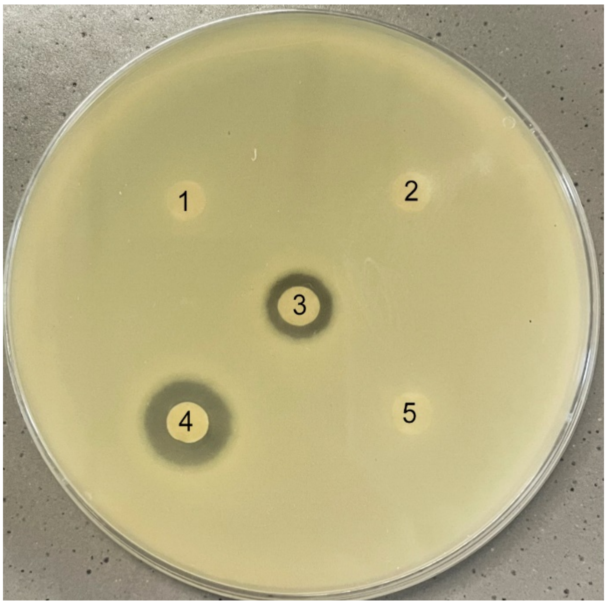

Figure S5. Susceptibility test of the thuricin CD variant (i.e. trnα' and trnβ') against *Bacillus cereus.* Spot 1: 5 μL 100 μM trnα'; Spot 2: 5 μL 100 μM trnβ'; Spot 3: 5 μL 100 μM trnα' + 5 μL 100 μM trnβ'; Spot 4: 5 μL 0.1 mg/ml kanamycin; Spot 5: 5 μL 30% isopropanol.

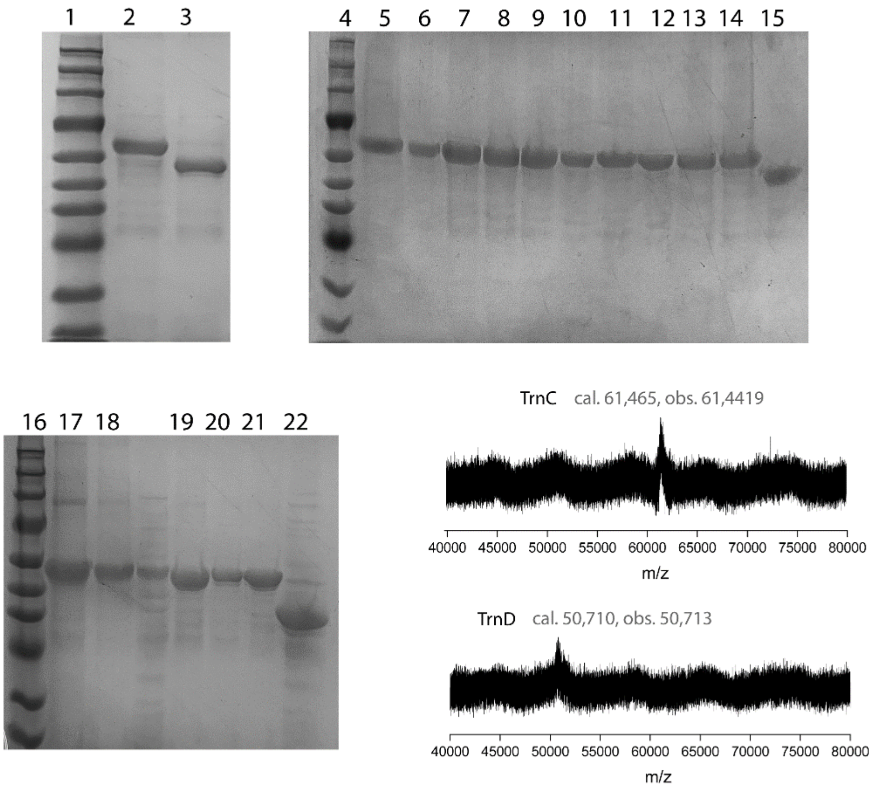

Figure S6. Characterization of the purified proteins, showing the SDS-PAGE of TrnC and TrnD and their mutants, and MALDI-MS spectra of TrnC and TrnD. Lanes 1, 4, 16 are protein makers (from bottom to top: 15 kD, 20 kD, 25 kD, 35 kD, 40 kD, 50 kD, 70 kD, 100 kD, 150 kD, 250 KD). Lane 2, wt-TrnC; lane 3, wt-TrnD; lane 5-15 are the R370A/K372A, C114A/C118A, C383A, C400A, C404A, C449A, C404A/C449A, C446A, C464A, C437A/C440A and ΔRRE mutants of TrnC; lane 19-25 are the E79A, C109A/C113A, C392A, C412A, C383A/C386A, and ΔRRE mutants of TrnD. MALDI-MS spectra show the exact molecular masses of TrnC and TrnD, which is within the margin of error.

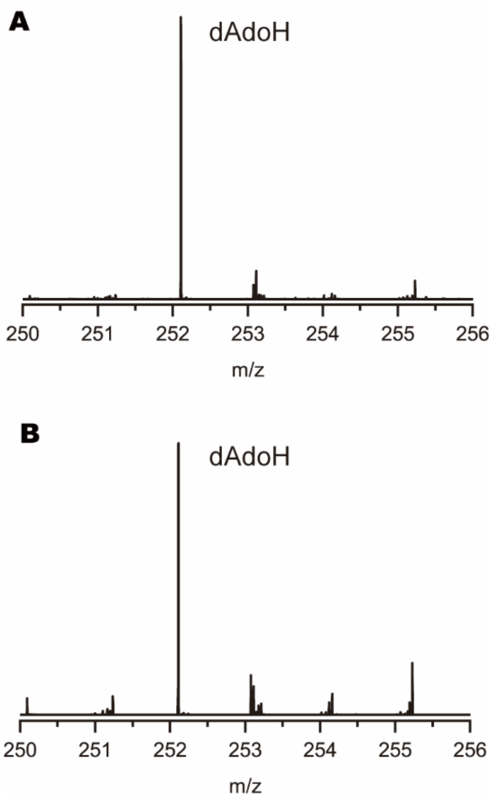

Figure S7. Characterization of the rSAM activity of TrnC and TrnD, showing the MS spectra of deoxyadenosine produced by (A) TrnC and (B) TrnD.

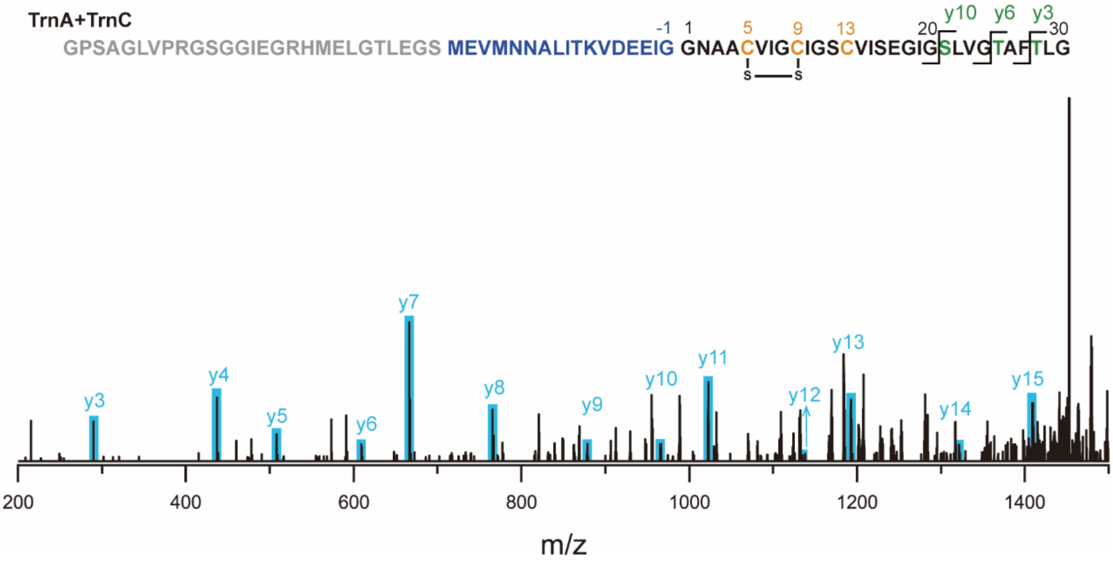

Figure S8. HR-MS/MS spectrum of the oxidized TrnA (with a disulfide bond) produced in the *in vitro* reaction with TrnC. We did not observe any mass shift for all the y ions, excluding the possibility of thioether crosslink formation.

y ions	Sequence	Calculated Mass $[M+H]^+$	Observed Mass	Mass Difference (Da)	Error (ppm)
y3	**T**LG	290.1710	290.1700	0.001	3.45
y4	F**T**LG	437.2395	437.2375	0.002	4.57
y5	AF**T**LG	508.2766	508.2757	0.0009	1.77
y6	**T**AF**T**LG	609.3243	609.3245	0.0002	0.33
y7	G**T**AF**T**LG	666.3457	666.3437	0.002	3.00
y8	VG**T**AF**T**LG	765.4141	765.4097	0.0044	5.75
y9	LVG**T**AF**T**LG	878.4982	878.4955	0.0027	3.07
y10	**S**LVG**T**AF**T**LG	965.5302	965.5245	0.0057	5.90
y11	G**S**LVG**T**AF**T**LG	1022.5517	1022.5469	0.0048	4.69
y12	IG**S**LVG**T**AF**T**LG	1135.6358	1135.6317	0.0041	3.61
y13	GIG**S**LVG**T**AF**T**LG	1192.6572	1192.6542	0.003	2.52
y14	EGIG**S**LVG**T**AF**T**LG	1321.6998	1321.7019	0.0021	1.59
y15	SEGIG**S**LVG**T**AF**T**LG	1408.7318	1408.7276	0.0042	2.98
y16	ISEGIG**S**LVG**T**AF**T**LG	1521.8159	NA	NA	NA

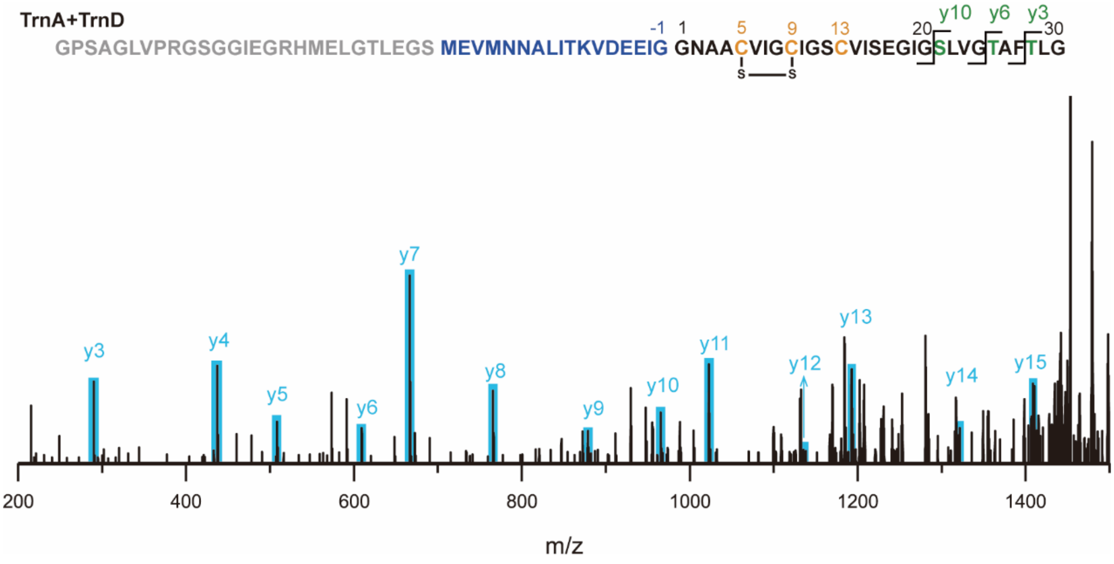

Figure S9. HR-MS/MS spectrum of the oxidized TrnA (with a disulfide bond) produced in the *in vitro* reaction with TrnD. We did not observe any mass shift for all the y ions, excluding the possibility of thioether crosslink formation.

y ions	Sequence	Calculated Mass [M+H]$^+$	Observed Mass	Mass Difference (Da)	Error (ppm)
y3	**T**LG	290.171	290.1711	0.0001	0.34
y4	F**T**LG	437.2395	437.2409	0.0014	3.20
y5	AF**T**LG	508.2766	508.2753	0.0013	2.56
y6	**T**AF**T**LG	609.3243	609.3228	0.0015	2.46
y7	G**T**AF**T**LG	666.3457	666.3466	0.0009	1.35
y8	VG**T**AF**T**LG	765.4141	765.4179	0.0038	4.96
y9	LVG**T**AF**T**LG	878.4982	878.4948	0.0034	3.87
y10	**S**LVG**T**AF**T**LG	965.5302	965.5338	0.0036	3.73
y11	G**S**LVG**T**AF**T**LG	1022.5517	1022.5465	0.0052	5.09
y12	IG**S**LVG**T**AF**T**LG	1135.6358	1135.6364	0.0006	0.53
y13	GIG**S**LVG**T**AF**T**LG	1192.6572	1192.6604	0.0032	2.68
y14	EGIG**S**LVG**T**AF**T**LG	1321.6998	1321.6925	0.0073	5.52
y15	SEGIG**S**LVG**T**AF**T**LG	1408.7318	1408.7333	0.0015	1.06
y16	ISEGIG**S**LVG**T**AF**T**LG	1521.8159	1521.8234	0.0075	4.93

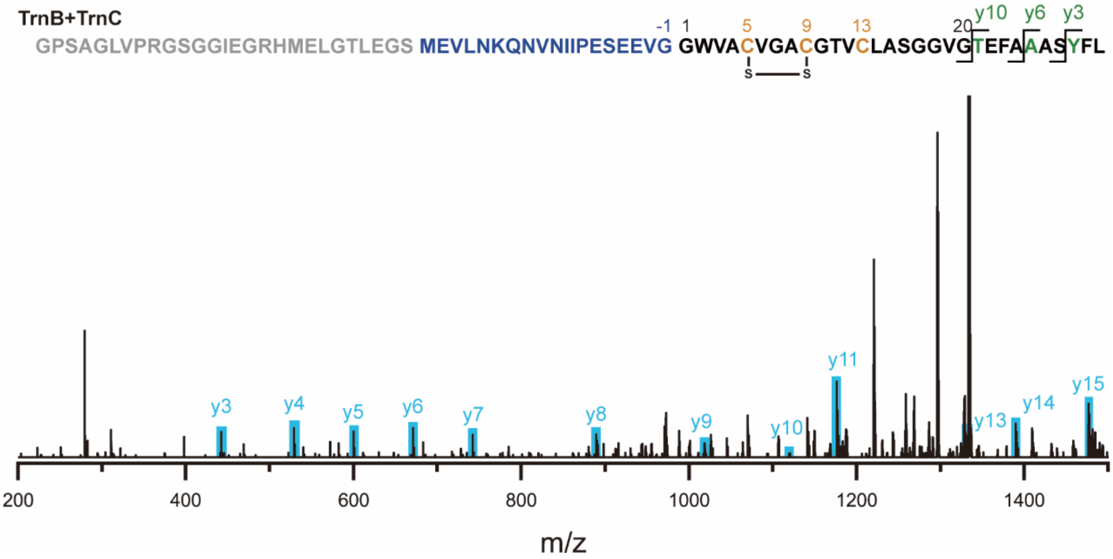

Figure S10. HR-MS/MS spectrum of the oxidized TrnB (with a disulfide bond) produced in the *in vitro* reaction with TrnC. We did not observe any mass shift for all the y ions, excluding the possibility of thioether crosslink formation.

y ions	Sequence	Calculated Mass $[M+H]^+$	Observed Mass	Mass Difference (Da)	Error (ppm)
y3	**Y**FL	442.2336	442.2324	0.0012	2.71
y4	S**Y**FL	529.2657	529.2623	0.0034	6.42
y5	AS**Y**FL	600.3028	600.3014	0.0014	2.33
y6	**A**AS**Y**FL	671.3399	671.3368	0.0031	4.62
y7	A**A**AS**Y**FL	742.377	742.3754	0.0016	2.16
y8	FA**A**AS**Y**FL	889.4454	889.4457	0.0003	0.34
y9	EFA**A**AS**Y**FL	1018.488	1018.4868	0.0012	1.18
y10	**T**EFA**A**AS**Y**FL	1119.5357	1119.5365	0.0008	0.71
y11	G**T**EFA**A**AS**Y**FL	1176.5572	1176.5527	0.0045	3.82
y12	VG**T**EFA**A**AS**Y**FL	1275.6256	1275.623	0.0026	2.04
y13	GVG**T**EFA**A**AS**Y**FL	1332.647	1332.6516	0.0046	3.45
y14	GGVG**T**EFA**A**AS**Y**FL	1389.6685	1389.6606	0.0079	5.68
y15	SGGVG**T**EFA**A**AS**Y**FL	1476.7005	1476.7078	0.0073	4.94
y16	ASGGVG**T**EFA**A**AS**Y**FL	1547.7377	1547.7386	0.0009	0.58

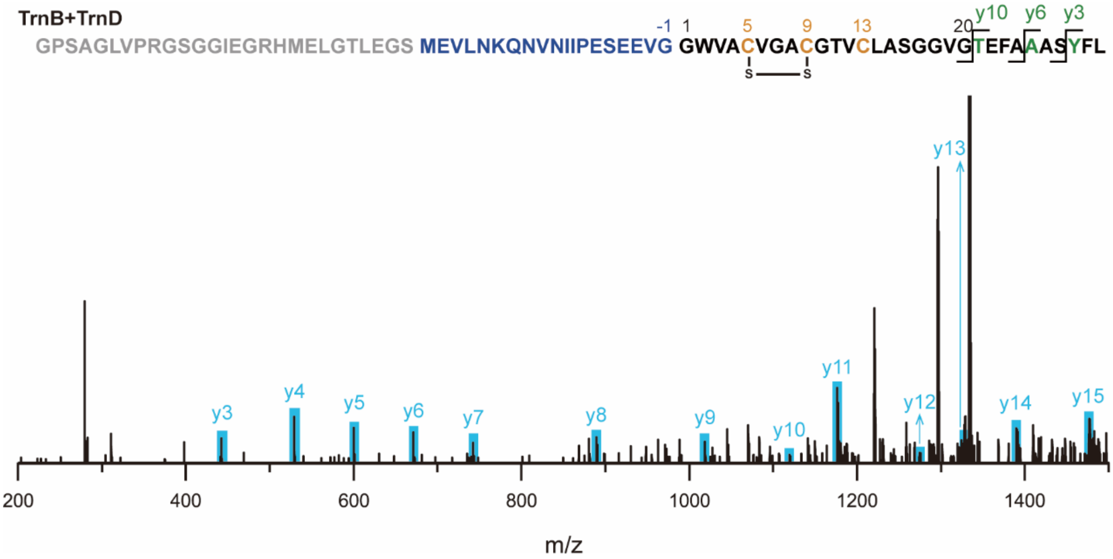

Figure S11. HR-MS/MS spectrum of the oxidized TrnB (with a disulfide bond) produced in the *in vitro* reaction with TrnD. We did not observe any mass shift for all the y ions, excluding the possibility of thioether crosslink formation.

y ions	Sequence	Calculated Mass $[M+H]^+$	Observed Mass	Mass Difference (Da)	Error (ppm)
y3	**Y**FL	442.2336	442.2310	0.0026	5.88
y4	S**Y**FL	529.2657	529.2655	0.0002	0.38
y5	AS**Y**FL	600.3028	600.3005	0.0023	3.83
y6	**A**AS**Y**FL	671.3399	671.3379	0.002	2.98
y7	A**A**AS**Y**FL	742.3770	742.3743	0.0027	3.64
y8	FA**A**AS**Y**FL	889.4454	889.4436	0.0018	2.02
y9	EFA**A**AS**Y**FL	1018.4880	1018.4819	0.0061	5.99
y10	**T**EFA**A**AS**Y**FL	1119.5357	1119.5332	0.0025	2.23
y11	G**T**EFA**A**AS**Y**FL	1176.5572	1176.5613	0.0041	3.48
y12	VG**T**EFA**A**AS**Y**FL	1275.6256	1275.6288	0.0032	2.51
y13	GVG**T**EFA**A**AS**Y**FL	1332.6470	1332.6515	0.0045	3.38
y14	GGVG**T**EFA**A**AS**Y**FL	1389.6685	1389.6737	0.0052	3.74
y15	SGGVG**T**EFA**A**AS**Y**FL	1476.7005	1476.6936	0.0069	4.67
y16	ASGGVG**T**EFA**A**AS**Y**FL	1547.7377	1547.7295	0.0082	5.30

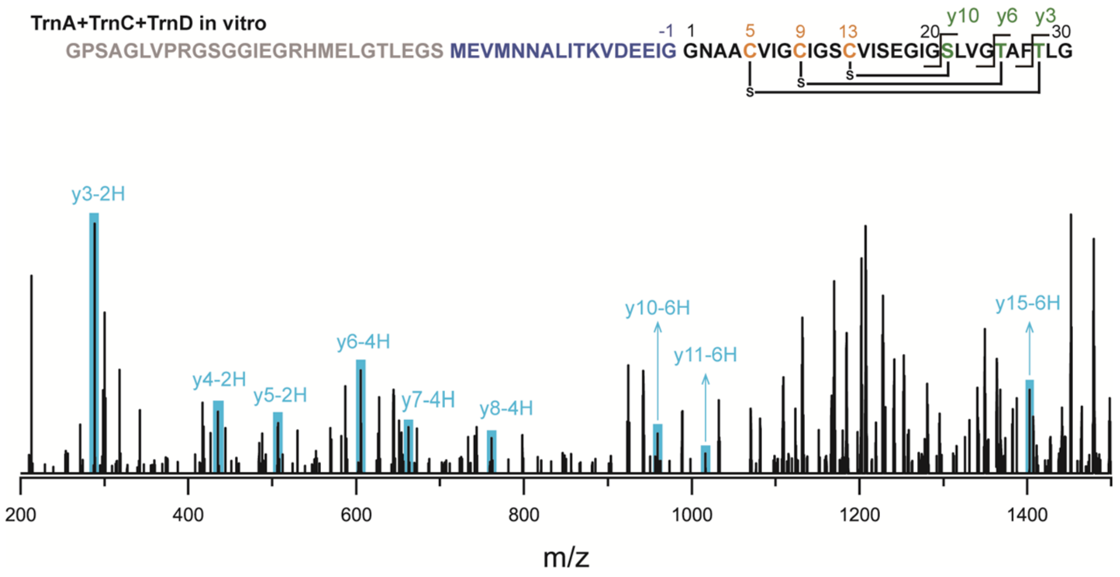

Figure S12. HR-MS/MS spectrum of the t3-TrnA produced in the in vitro reaction with TrnC and TrnD.

y ions	Sequence	Calculated Mass $[M+H]^+$	Observed Mass	Mass Difference (Da)	Error (ppm)
y3	**T**LG	290.1710	288.1545	2.0165	2.95
y4	F**T**LG	437.2395	435.2236	2.0159	0.57
y5	AF**T**LG	508.2766	506.2602	2.0164	1.48
y6	**T**AF**T**LG	609.3243	605.2925	4.0318	0.83
y7	G**T**AF**T**LG	666.3457	662.3129	4.0328	2.26
y8	VG**T**AF**T**LG	765.4141	761.3815	4.0326	1.71
y9	LVG**T**AF**T**LG	878.4982	NA	NA	NA
y10	**S**LVG**T**AF**T**LG	965.5302	959.4792	6.0510	4.22
y11	G**S**LVG**T**AF**T**LG	1022.5517	1016.5078	6.0439	3.00
y12	IG**S**LVG**T**AF**T**LG	1135.6358	NA	NA	NA
y13	GIG**S**LVG**T**AF**T**LG	1192.6572	NA	NA	NA
y14	EGIG**S**LVG**T**AF**T**LG	1321.6998	NA	NA	NA
y15	SEGIG**S**LVG**T**AF**T**LG	1408.7318	1402.6816	6.0502	2.32
y16	ISEGIG**S**LVG**T**AF**T**LG	1521.8159	NA	NA	NA

y ions of -2 Da, -4 Da, and -6 Da are highlighted by yellow, blue, and green, respectively.

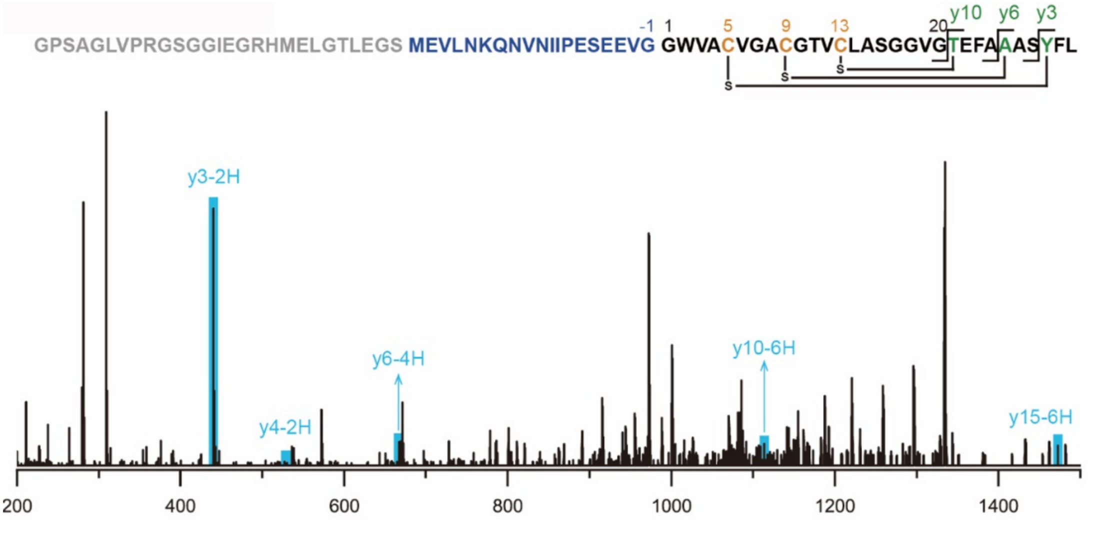

Figure S13. HR-MS/MS spectrum of the t3-TrnB produced in the in vivo reaction with TrnC and TrnD.

y ions	Sequence	Calculated Mass [M+H]$^+$	Observed Mass	Mass Difference (Da)	Error (ppm)
y3	**Y**FL	442.2336	440.2168	2.0168	2.61
y4	S**Y**FL	529.2657	527.2507	2.0150	1.23
y5	AS**Y**FL	600.3028	NA	NA	NA
y6	**A**AS**Y**FL	671.3399	667.3075	4.0324	1.65
y7	A**A**AS**Y**FL	742.377	NA	NA	NA
y8	FA**A**AS**Y**FL	889.4454	NA	NA	NA
y9	EFA**A**AS**Y**FL	1018.488	NA	NA	NA
y10	**T**EFA**A**AS**Y**FL	1119.5357	1113.4918	6.0439	2.74
y11	G**T**EFA**A**AS**Y**FL	1176.5572	NA	NA	NA
y12	VG**T**EFA**A**AS**Y**FL	1275.6256	NA	NA	NA
y13	GVG**T**EFA**A**AS**Y**FL	1332.647	NA	NA	NA
y14	GGVG**T**EFA**A**AS**Y**FL	1389.6685	NA	NA	NA
y15	SGGVG**T**EFA**A**AS**Y**FL	1476.7005	1470.6491	6.0514	3.03
y16	ASGGVG**T**EFA**A**AS**Y**FL	1547.7377	NA	NA	NA

y ions of -2 Da, -4 Da, and -6 Da are highlighted by yellow, blue, and green, respectively.

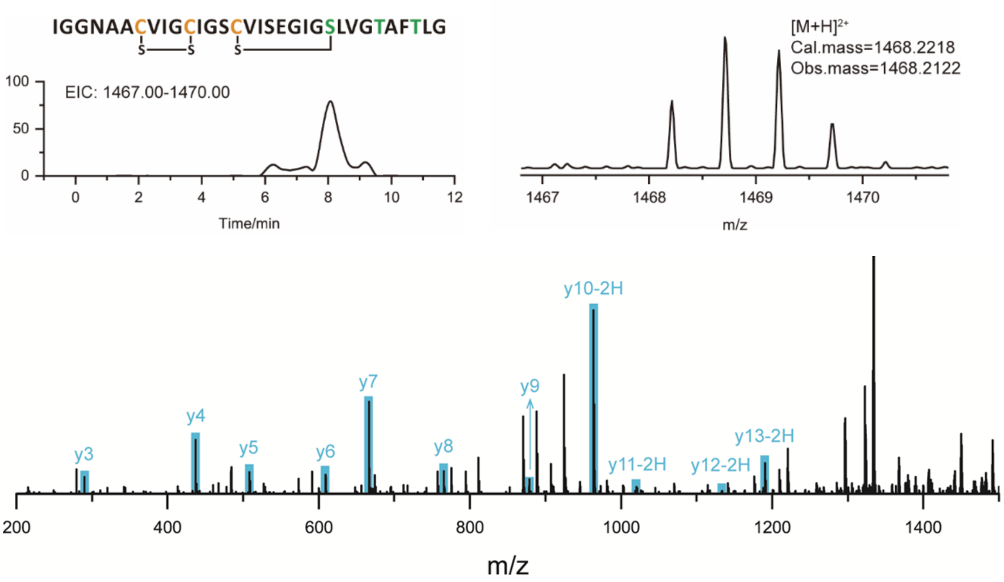

Figure S14. HR-MS and HR-MS/MS analysis of the GluC-digested fragment of the -4 Da product of TrnA (i.e. t1-TrnA$_{(-2\text{-}30)}$(-2H)) produced in the reaction with TrnC *in vitro*. HR-MS/MS analysis showed the thioether crosslink is formed between Cys13 and Ser21. This is consistent with reaction directionality analysis, showing the thioether crosslink formation on TrnA proceeds from Cys13 to Cys5 in a C-to-N manner (Figure 3 in the main text).

**y ions**	**Sequence**	**Calculated Mass [M+H]$^+$**	**Observed Mass**	**Mass Difference (Da)**	**Error (ppm)**
y3	**T**LG	290.1710	290.1702	0.0008	2.76
y4	F**T**LG	437.2395	437.2378	0.0017	3.89
y5	AF**T**LG	508.2766	508.2756	0.0010	1.97
y6	**T**AF**T**LG	609.3243	609.3227	0.0016	2.63
y7	G**T**AF**T**LG	666.3457	666.3424	0.0033	4.95
y8	VG**T**AF**T**LG	765.4141	765.4102	0.0039	5.10
y9	LVG**T**AF**T**LG	878.4982	878.4976	0.0006	0.68
y10	**S**LVG**T**AF**T**LG	965.5302	963.5093	2.0209	5.45
y11	G**S**LVG**T**AF**T**LG	1022.5517	1020.5327	2.0190	3.28
y12	IG**S**LVG**T**AF**T**LG	1135.6358	1133.6173	2.0185	2.51
y13	GIG**S**LVG**T**AF**T**LG	1192.6572	1190.6364	2.0208	4.33
y14	EGIG**S**LVG**T**AF**T**LG	1321.6998	NA	NA	NA
y15	SEGIG**S**LVG**T**AF**T**LG	1408.7318	NA	NA	NA
y16	ISEGIG**S**LVG**T**AF**T**LG	1521.8159	NA	NA	NA

y ions of -2 Da are highlighted by yellow.

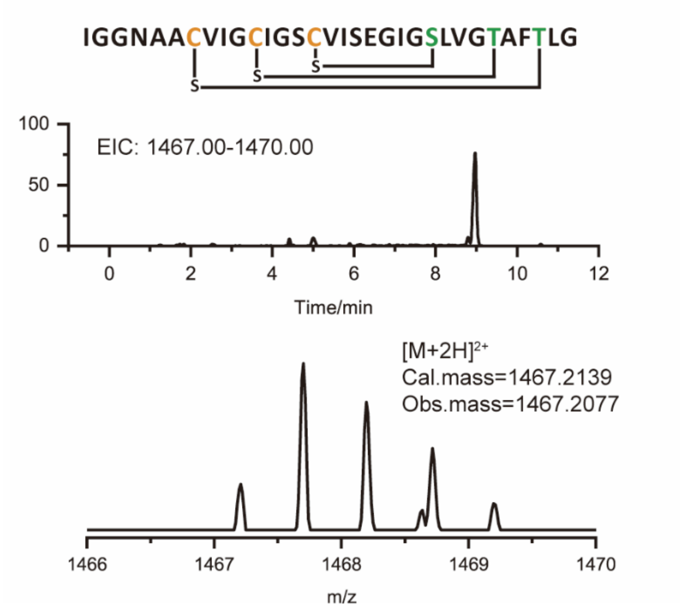

Figure S15. Production of fully modified TrnA (t3-TrnA) using an increased concentration (100 μM) of TrnC in vitro, showing the LC-HRMS data of the GluC-digested fragment of t3-TrnA.

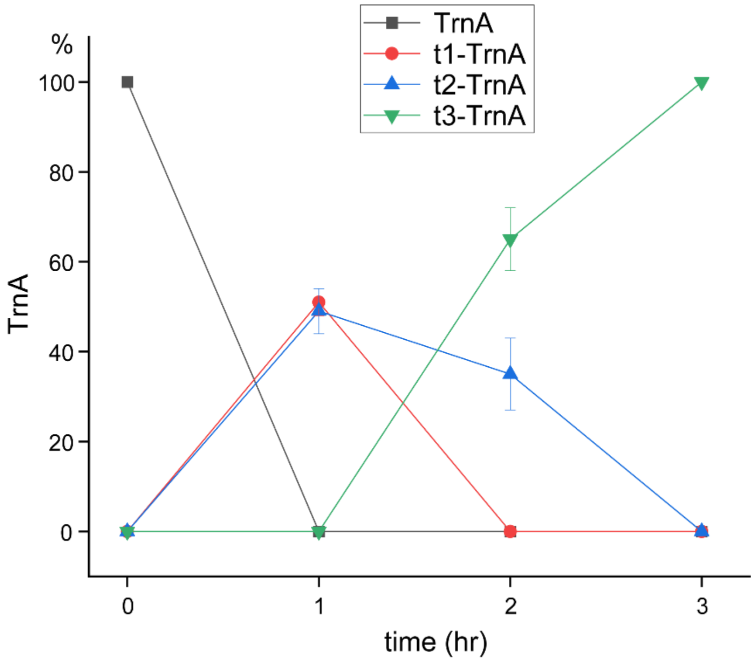

Figure S16. Time course analysis of TrnA modification by the combined action of TrnC and TrnD. Quantitative analysis was performed according to the MS intensities of the corresponding species carrying different thioether crosslinks, as detailed in Figure S22.

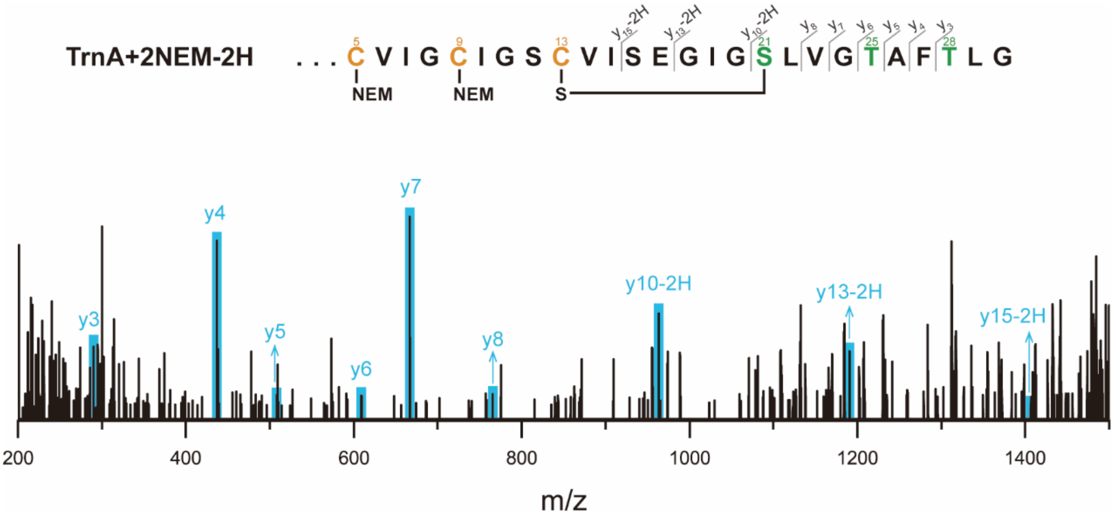

Figure S17. HR-MS/MS spectrum of the NEM-derivatized t1-TrnA shown in Figure 3B in the main text.

y ions	Sequence	Calculated Mass $[M+H]^+$	Observed Mass	Mass Difference (Da)	Error (ppm)
y3	**T**LG	290.1710	290.1711	0.0001	0.34
y4	F**T**LG	437.2395	437.2411	0.0016	3.66
y5	AF**T**LG	508.2766	508.2752	0.0014	2.75
y6	**T**AF**T**LG	609.3243	609.3277	0.0034	5.58
y7	G**T**AF**T**LG	666.3457	666.3454	0.0003	0.45
y8	VG**T**AF**T**LG	765.4141	765.4106	0.0035	4.57
y9	LVG**T**AF**T**LG	878.4982	NA	NA	NA
y10	**S**LVG**T**AF**T**LG	965.5302	963.5156	2.0146	1.09
y11	G**S**LVG**T**AF**T**LG	1022.5517	NA	NA	NA
y12	IG**S**LVG**T**AF**T**LG	1135.6358	NA	NA	NA
y13	GIG**S**LVG**T**AF**T**LG	1192.6572	1190.6387	2.0185	2.39
y14	EGIG**S**LVG**T**AF**T**LG	1321.6998	NA	NA	NA
y15	SEGIG**S**LVG**T**AF**T**LG	1408.7318	1406.7243	2.0075	5.79
y16	ISEGIG**S**LVG**T**AF**T**LG	1521.8159	NA	NA	NA

y ions of -2 Da are highlighted by yellow.

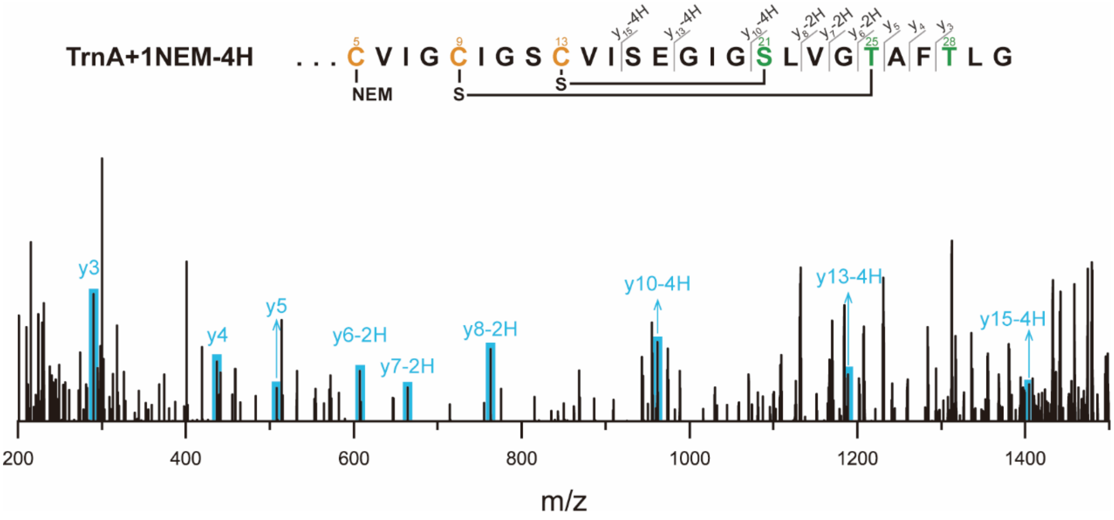

Figure S18. HR-MS/MS spectrum of the NEM-derivatized t2-TrnA shown in Figure 3B in the main text.

y ions	Sequence	Calculated Mass [M+H]$^+$	Observed Mass	Mass Difference (Da)	Error (ppm)
y3	**T**LG	290.1710	290.1712	0.0002	0.69
y4	F**T**LG	437.2395	437.2371	0.0024	5.49
y5	AF**T**LG	508.2766	508.2796	0.0030	5.90
y6	**T**AF**T**LG	609.3243	607.3113	2.0130	4.36
y7	G**T**AF**T**LG	666.3457	664.3289	2.0168	1.73
y8	VG**T**AF**T**LG	765.4141	763.3948	2.0193	4.78
y9	LVG**T**AF**T**LG	878.4982	NA	NA	NA
y10	**S**LVG**T**AF**T**LG	965.5302	961.5019	4.0283	3.12
y11	G**S**LVG**T**AF**T**LG	1022.5517	NA	NA	NA
y12	IG**S**LVG**T**AF**T**LG	1135.6358	NA	NA	NA
y13	GIG**S**LVG**T**AF**T**LG	1192.6572	1188.6216	4.0356	3.62
y14	EGIG**S**LVG**T**AF**T**LG	1321.6998	NA	NA	NA
y15	SEGIG**S**LVG**T**AF**T**LG	1408.7318	1404.6950	4.0368	3.92
y16	ISEGIG**S**LVG**T**AF**T**LG	1521.8159	1517.7795	4.0364	3.36

y ions of -2 Da and -4 Da are highlighted by yellow and blue, respectively.

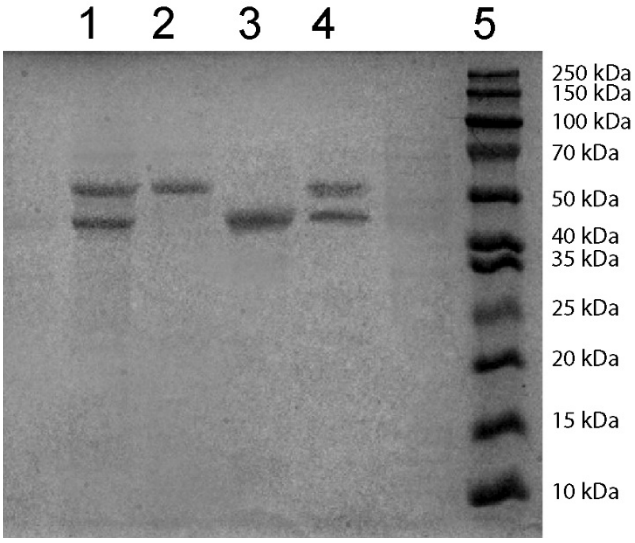

Figure S19. Pull down analysis of TrnC and TrnD. Lane 1, His-Tagged TrnC and non-Tagged TrnD obtained by expression of the pRSFdue-His$_6$-TrnC-TrnD construct; Lane 2, His-Tagged TrnC obtained by expression of the pET28-derived construct (pET28a-TrnC); Lane 3, His-Tagged TrnD obtained by expression of the pET28-derived construct (pET28a-TrnD); 4, His-Tagged TrnD and non-Tagged TrnC, which obtained by purification of the mixture in which the cell lysates expressing His-Tagged TrnD (pET28a-TrnD) was mixed with the cell lysate expressing non-Tagged TrnC (pRSFduet-TrnD). Lane 5, protein molecular marker.

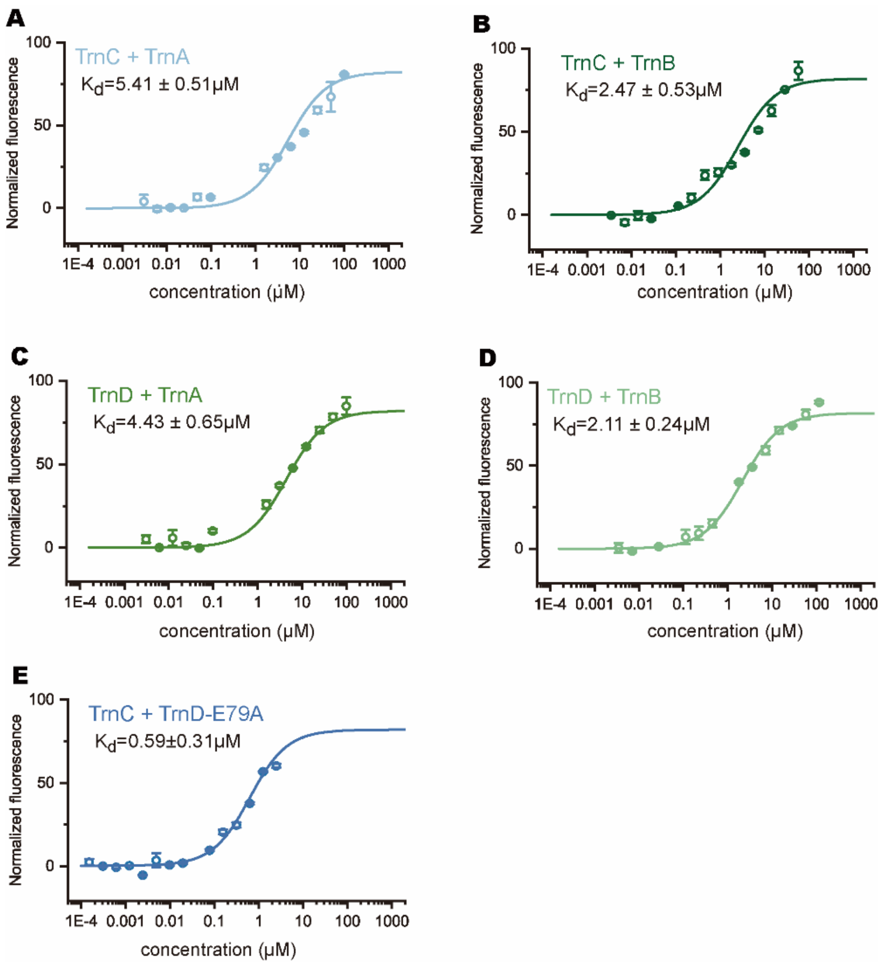

Figure S20. MST analysis, showing fluorescently labeled TrnC with (A) TrnA and (B) TrnB, and fuorescently labeled TrnD with (C) TrnA and (D) TrnB, and (E) fluorescently labeled TrnC with the E79A mutant of TrnD.

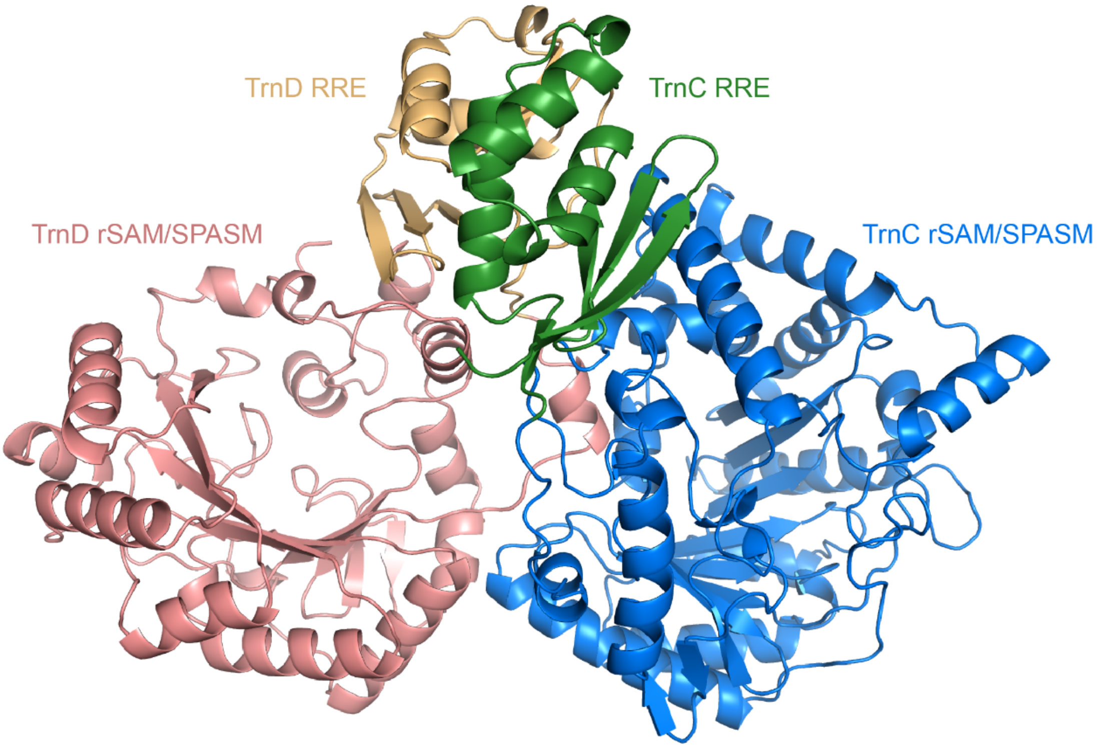

Figure S21. Alphafold structure of the binary complex of TrnC and TrnD. The heterodimeric complex is formed primarily through the interaction between the two RRE domains. The predicted template modeling (pTM) score and the interface predicted template modeling (ipTM) score are 0.8 and 0.79, respectively.

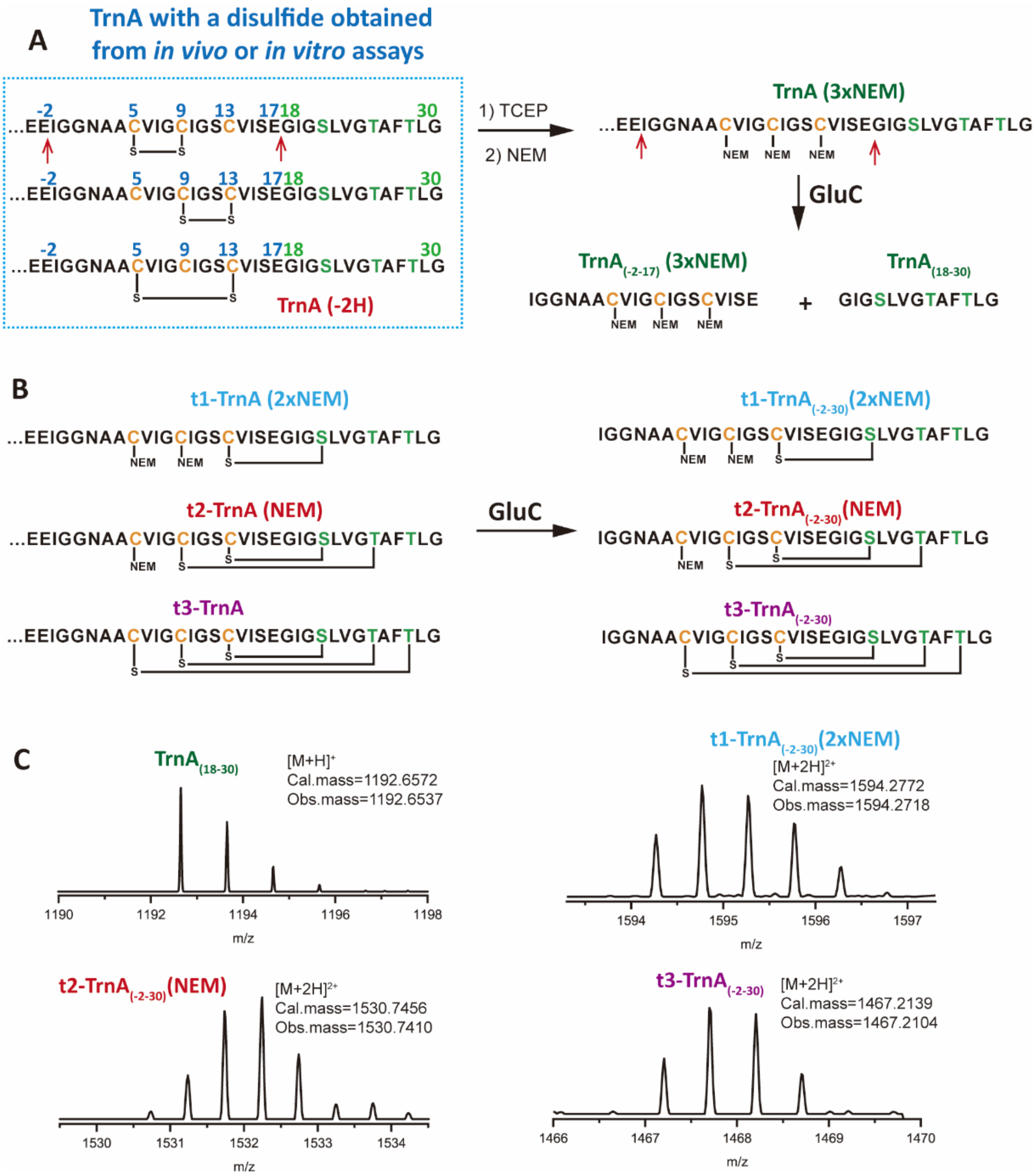

Figure S22. Schematic overview of the procedure for analyzing the in vitro or in vivo activities of TrnC and TrnD with the substrate TrnA. (A) In the absence of sactionine formation, GluC cleaves the peptide between E17 and G18, generating two fragments, $TrnA_{(-2-17)}$ and the unmodified fragment $TrnA_{(18-30)}$. (B) When one or more sactionine rings are formed, GluC cannot cleave at the site between E17 and G18, resulting in the production of a single fragment, $TrnA_{(-2-30)}$. Due to the NEM derivatization of different products, the fragments carrying distinct sactionine rings can be easily identified and quantified. (C) HR-MS spectra of the product corresponding to t0-TrnA (no thioether crosslink), t1-TrnA, t2-TrnA, and t3-TrnA.

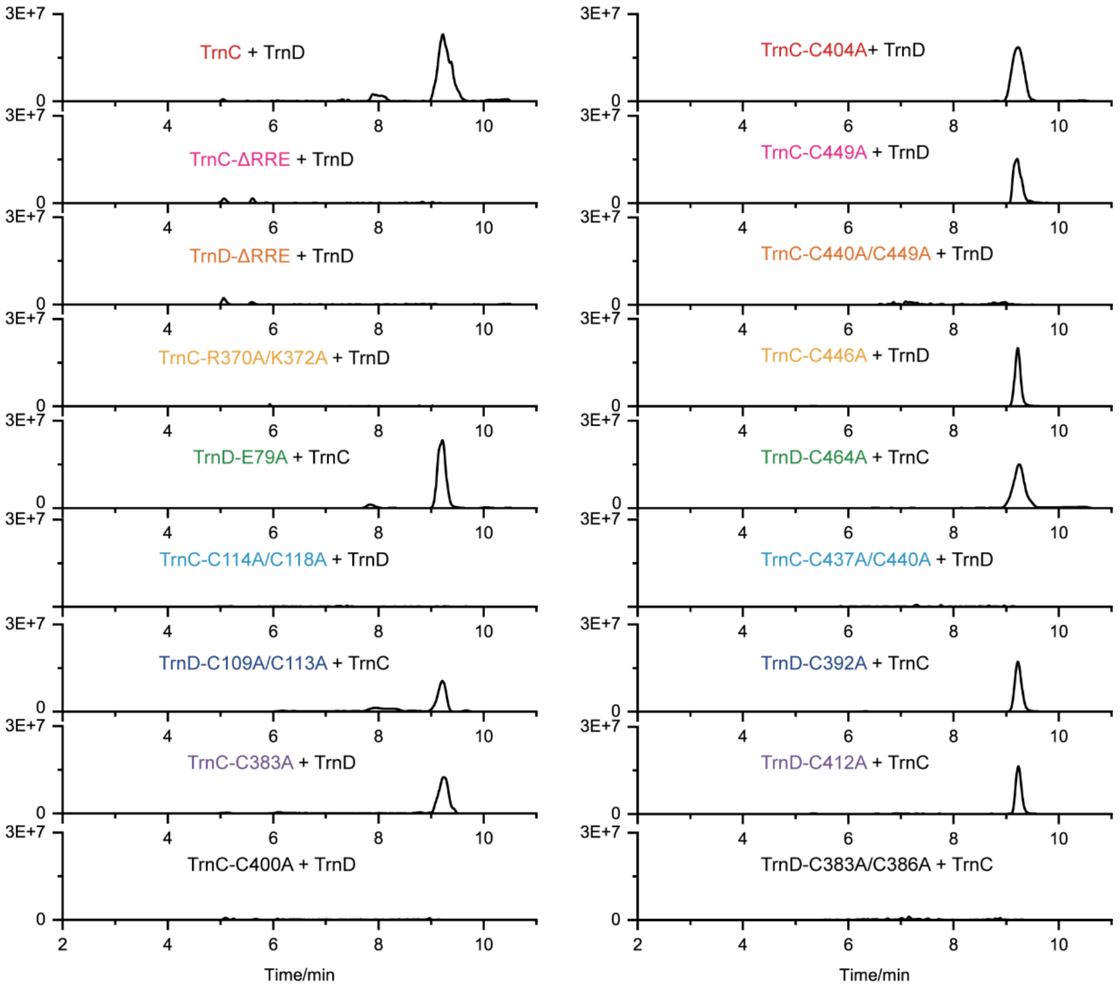

Figure S23. Production of t3-TrnA in the in vitro analysis, showing the extracted ion chromatograms of 1467.2 (corresponding to the $[M + 2H]^{2+}$ of t3-TrnA$_{(-2-30)}$) for assays using the wild type or mutant enzymes.

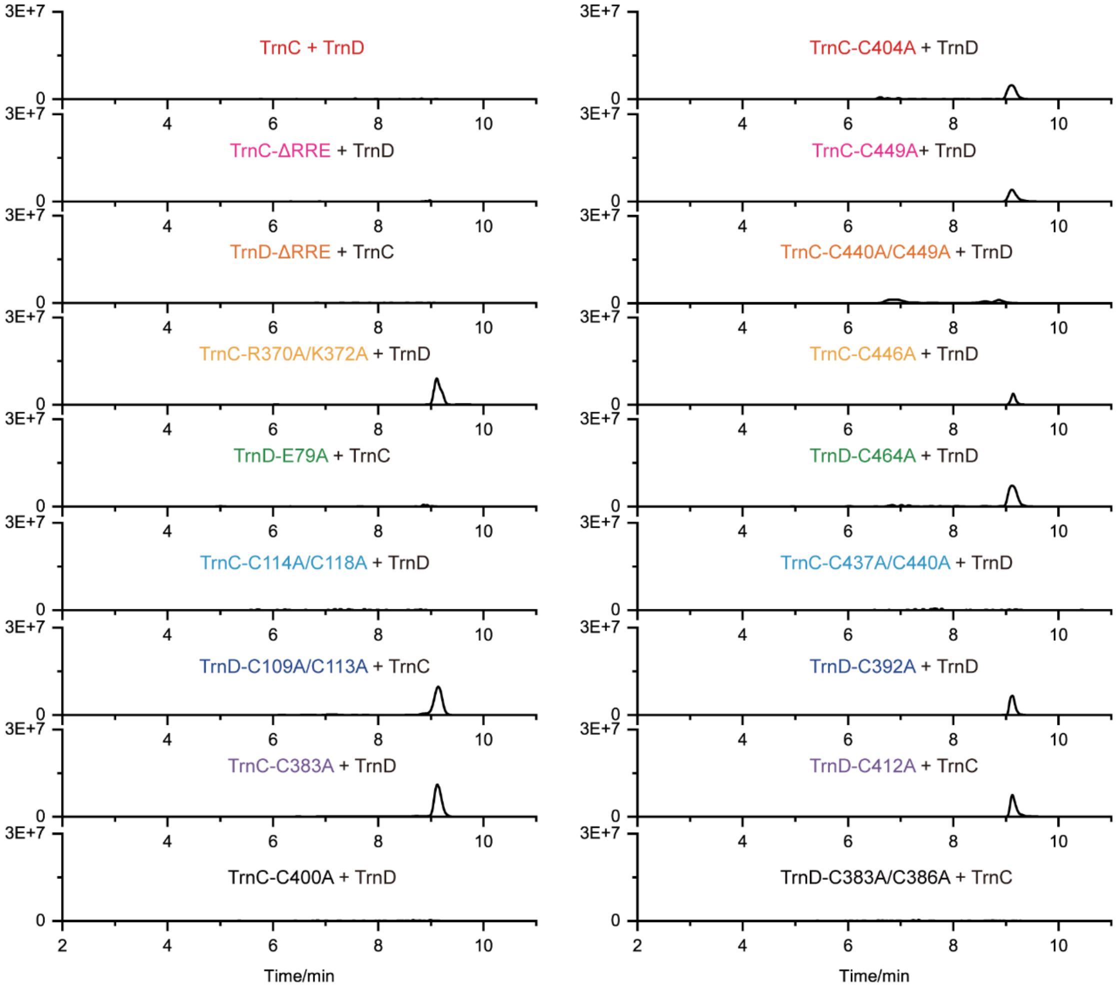

Figure S24. Production of t2-TrnA in the in vitro analysis, showing the extracted ion chromatograms of 1530.7 (corresponding to the $[M + 2H]^{2+}$ of t2-TrnA$_{(-2-30)}$(NEM) for assays using the wild type or mutant enzymes.

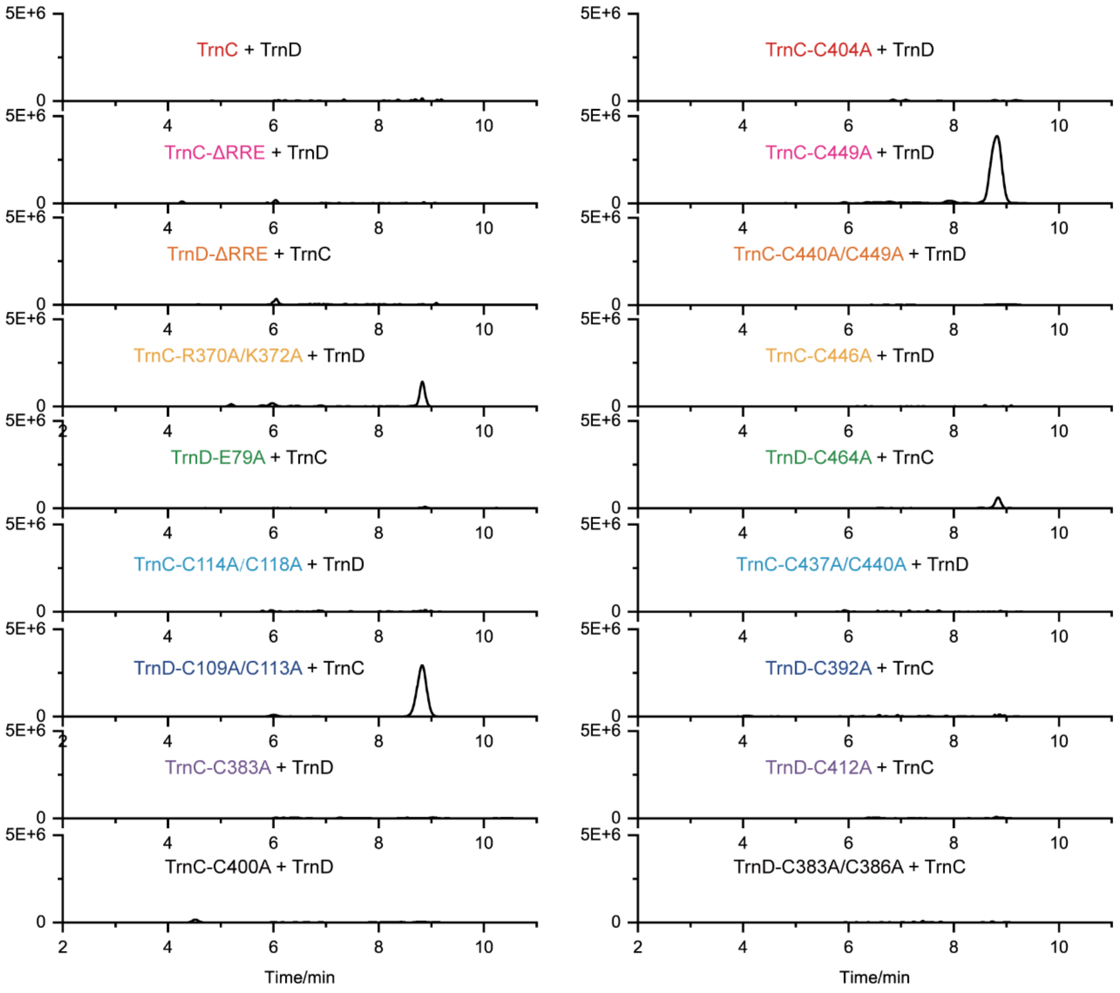

Figure S25. Production of t2-TrnA in the in vitro analysis, showing the extracted ion chromatograms of 1594.3(corresponding to the $[M + 2H]^{2+}$ of t1-TrnA$_{(-2-30)}$(2xNEM) for various assays using the wild type or mutant enzymes.

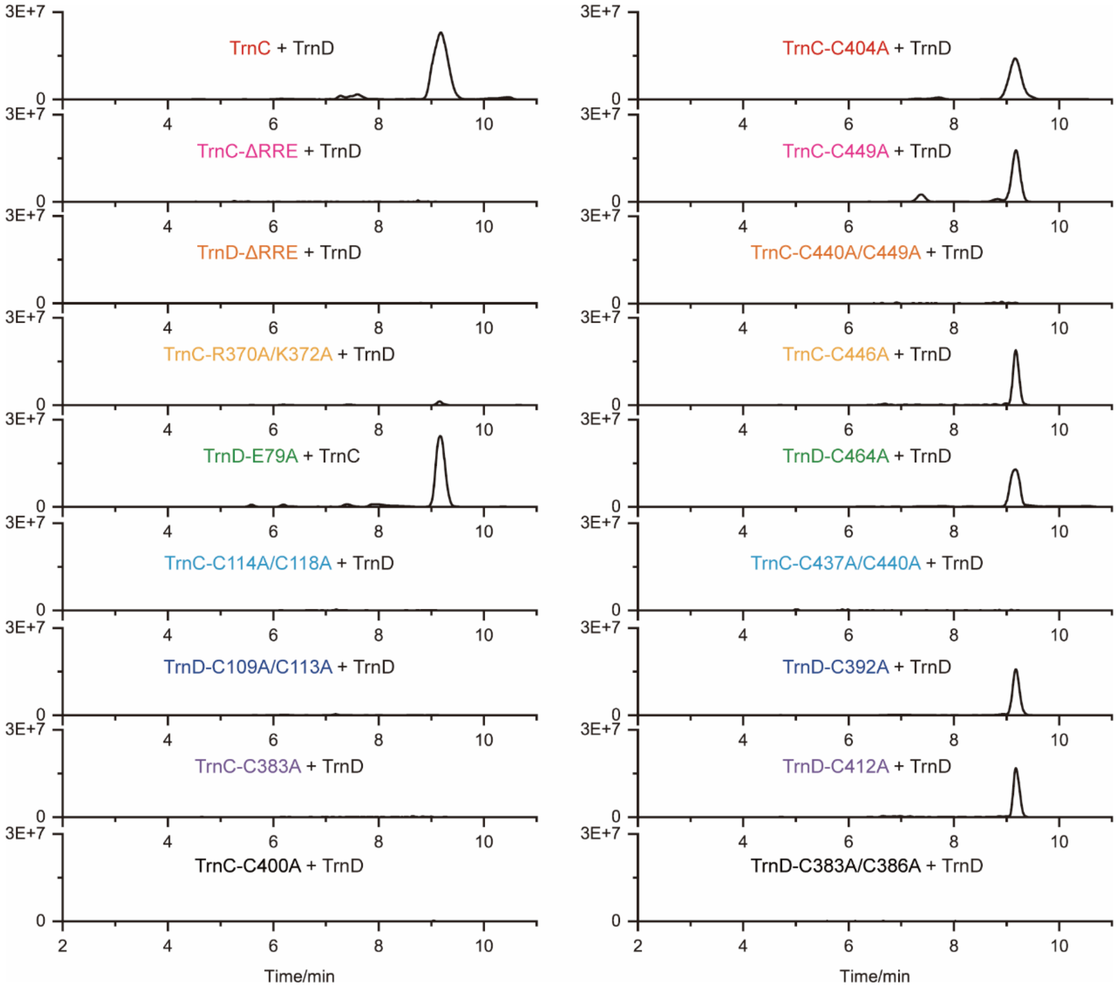

Figure S26. Production of t3-TrnB in the co-expression studies, showing the extracted ion chromatograms of 1059.2 (corresponding to the $[M + 2H]^{2+}$ of t3-TrnB$_{(-2\text{-}30)}$) for studies with the wild type or mutant enzymes.

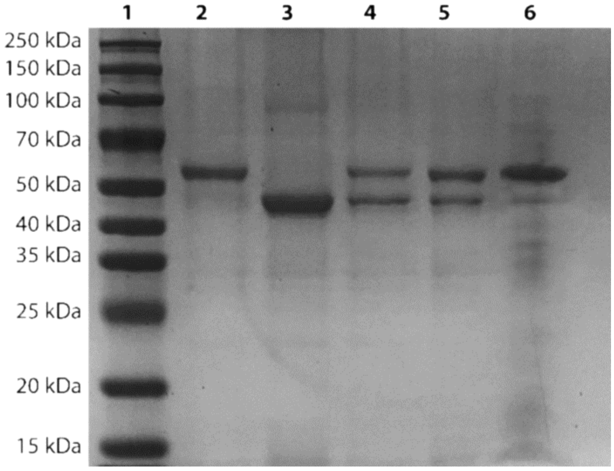

Figure S27. Pull down analysis of TrnC and TrnD proteins. Lane 1, protein molecular marker; lane 2, His-tagged TrnC; lane 3, His-tagged TrnD; lane 4, His-tagged TrnC and non-tagged TrnD obtained by expression of the pRSFdue-His_6-TrnC-TrnD construct; lane 5, His-tagged TrnC and non-tagged TrnD (E79A) obtained by expression of the pRSFdue-His_6-TrnC-TrnD-(E79A) construct; lane 6, His-tagged TrnC (R370A/K372A) and non-Tagged TrnD obtained by expression of the pRSFdue-His_6-TrnC-(R370A/K372A)-TrnD construct.

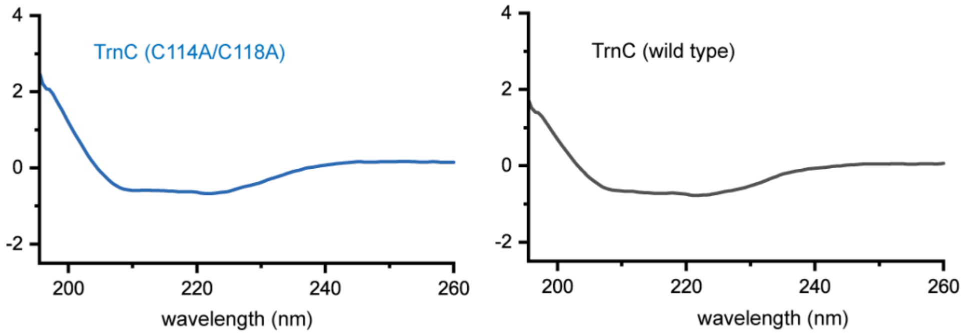

Figure S28. CD spectrum of the wild type and the C114A/C118A mutant of TrnC.

A

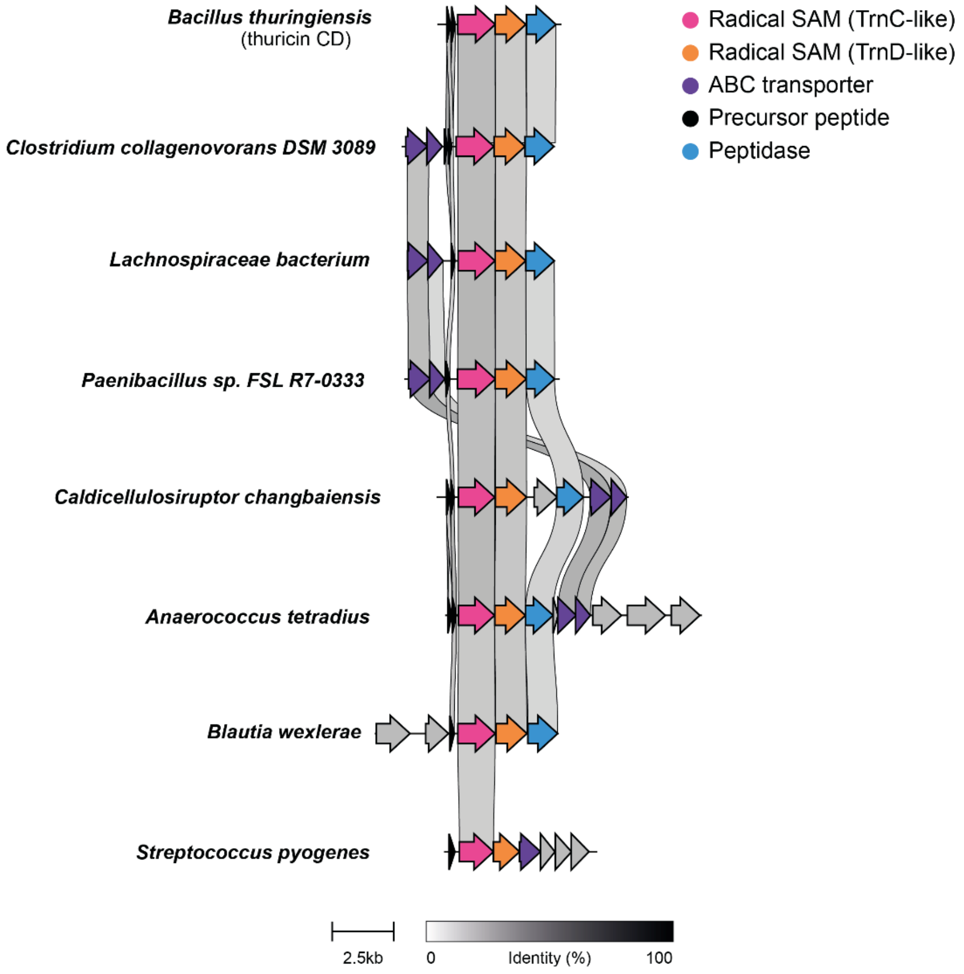

B

WP_098404387.1	100%
WP_072832080.1	50%
MBO4497714.1	54%
WP_076082151.1	57.5%
WP_164742554.1	45.93%
WP_276881943.1	44.26%
WP_195481260.1	46.13%
HES7786119.1	37.31%
WP_255259242.1	40.08%
	WP_098404387.1

C

WP_098404386.1	100%
WP_072832081.1	47.1%
MBO4497715.1	46.34%
WP_076082153.1	48.79%
WP_127350936.1	45.41%
WP_276881945.1	41.54%
WP_195481258.1	48.06%
HES7786120.1	33.71%
WP_240518687.1	32.67%
	WP_098404386.1

Figure S29. Genome mining of gene clusters similar to that for thuricin CD biosynthesis, showing (A) the gene clusters from various strains, and the sequence similarities (B) between TrnC (WP 098404387.1) and TrnC-like enzymes, and (C) between TrnD (WP 098404386.1) and TrnD-like enzymes.

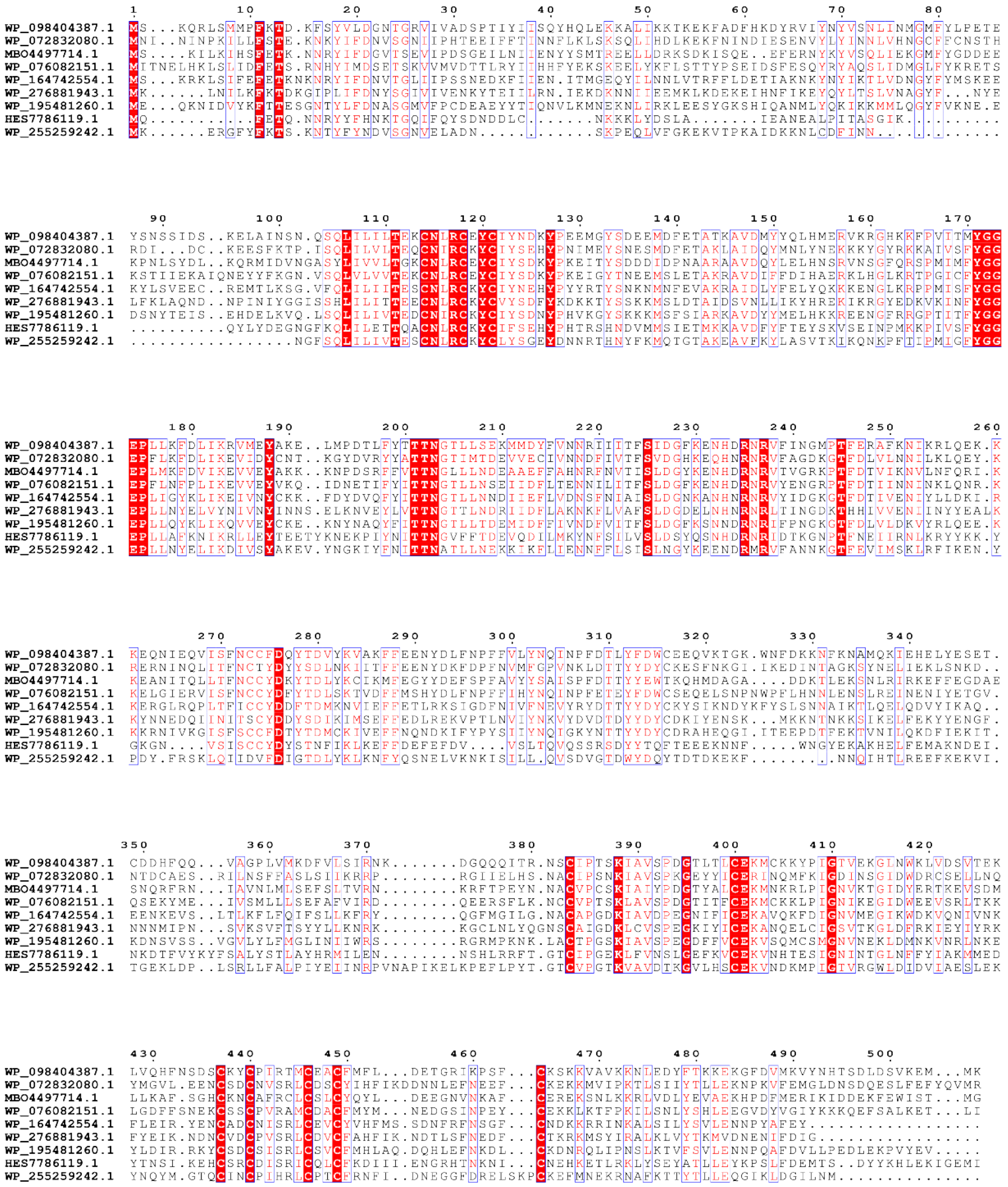

Figure S30. Multiple sequence alignment of TrnC (WP 098404387.1) with other TrnC-like enzymes obtained from genome mining.

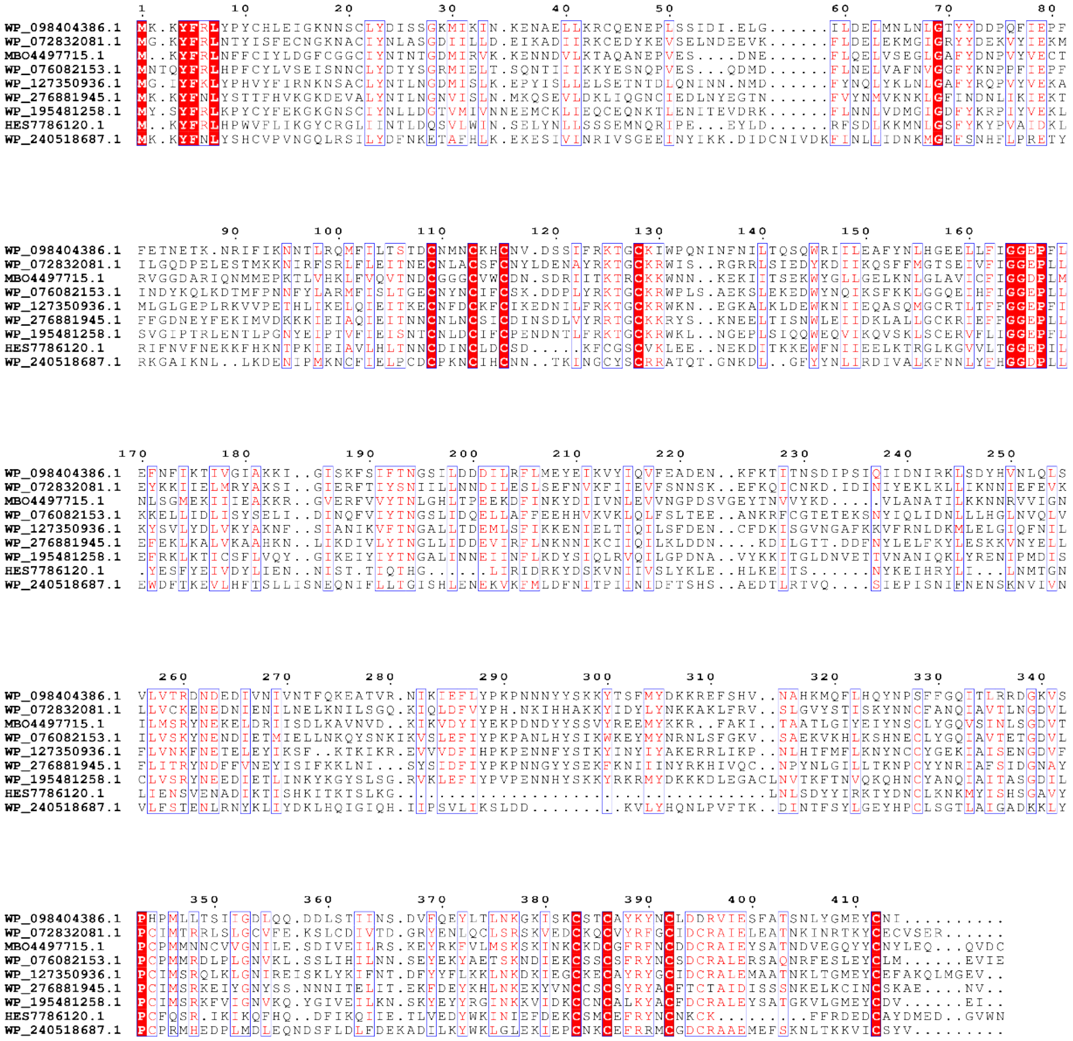

Figure S31. Multiple sequence alignment of TrnD (WP 098404386.1) with other TrnD-like enzymes obtained from genome mining.

A

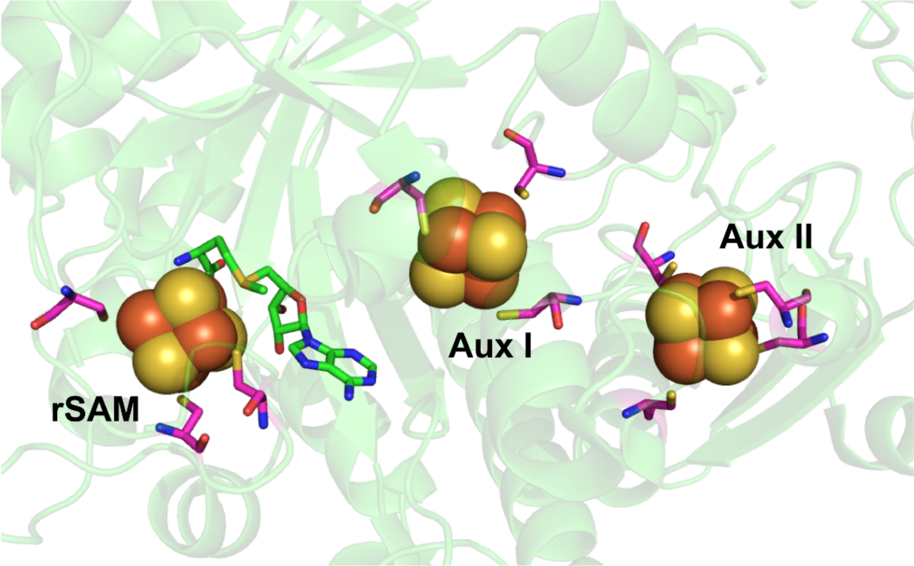

B

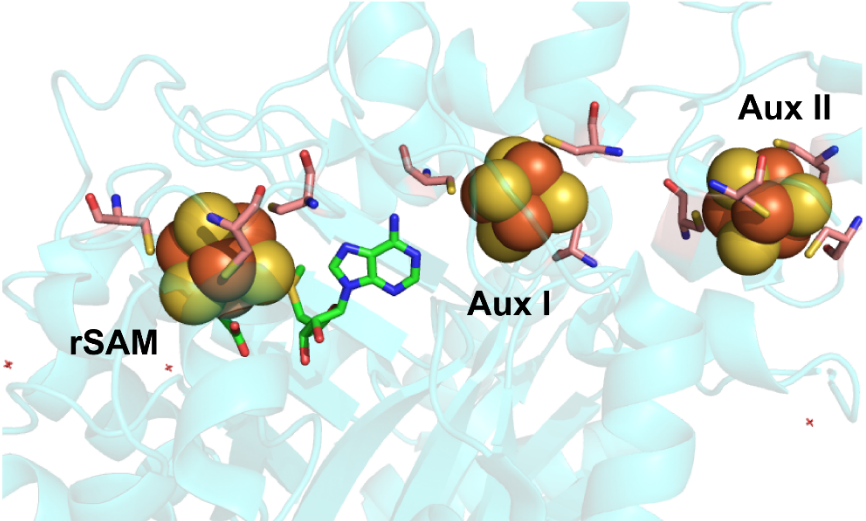

Figure S32. Crystal structure of (A) CetB(*15*) and (B) SuiB(*16*), showing overall structures and the Cys residues involved in binding of the [4Fe-4S] clusters.

Table S1. The sequences of the wilde type proteins expressed in this study. The $Cx_{9-15}Gx_4Cx_nCx_2Cx_5Cx_3Cx_nC$ motif in TrnC and the Cx_2Cx_5C motif in TrnD are shown in red.

>**pCold-TF-TrnA** (His$_6$-Trigger Factor-HRV 3C site-**TrnA**)<br>NHKVHHHHHHMQVSVETTQGLGRRVTITIAADSIETAVKSELVNVAKKVRIDGFRKGKVPMNIVAQRYGASVRQDVLGDLMSRNFIDAIIKEKINPAGAPTYVPGEYKLGEDFTYSVEFEVYPEVELQGLEAIEVEKPIVEVTDADVDGMLDTLRKQQATWKEKDGAVEAEDRVTIDFTGSVDGEEFEGGKASDFVLAMGQGRMIPGFEDGIKGHKAGEEFTIDVTFPEEYHAENLKGKAAKFAINLKKVEERELPELTAEFIKRFGVEDGSVEGLRAEVRKNMERELKSAIRNRVKSQAIEGLVKANDIDVPAALIDSEIDVLRRQAAQRFGGNEKQALELPRELFEEQAKRRVVVGLLLGEVIRTNELKADEERVKGLIEEMASAYEDPKEVIEFYSKNKELMDNMRNVALEEQAVEAVLAKAKVTEKETTFNELMNQQASAGLEVLFQGPSAGLVPRGSGGIEGRHMELGTLEGS**MEVMNNALITKVDEEIGGNAACVIGCIGSCVISEGIGSLVGTAFTLG**
>**pCold-TF-TrnB** (His$_6$-Trigger Factor-HRV 3C site-**TrnB**)<br>NHKVHHHHHHMQVSVETTQGLGRRVTITIAADSIETAVKSELVNVAKKVRIDGFRKGKVPMNIVAQRYGASVRQDVLGDLMSRNFIDAIIKEKINPAGAPTYVPGEYKLGEDFTYSVEFEVYPEVELQGLEAIEVEKPIVEVTDADVDGMLDTLRKQQATWKEKDGAVEAEDRVTIDFTGSVDGEEFEGGKASDFVLAMGQGRMIPGFEDGIKGHKAGEEFTIDVTFPEEYHAENLKGKAAKFAINLKKVEERELPELTAEFIKRFGVEDGSVEGLRAEVRKNMERELKSAIRNRVKSQAIEGLVKANDIDVPAALIDSEIDVLRRQAAQRFGGNEKQALELPRELFEEQAKRRVVVGLLLGEVIRTNELKADEERVKGLIEEMASAYEDPKEVIEFYSKNKELMDNMRNVALEEQAVEAVLAKAKVTEKETTFNELMNQQASAGLEVLFQGPSAGLVPRGSGGIEGRHMELGTLEGS**MEVLNKQNVNIIPESEEVGGWVACVGACGTVCLASGGVGTEFAAASYFL**
>**pET28a-TrnC** (His$_6$-**TrnC**)<br>GSSHHHHHHSSGLVPRGSH**MSKKRLSMMPFKTDKFSYALDGNTGRVIVADKPTLYIISHFHKFEKEELLKKTKGKFAELHQDYLTTYNYVSSLINMGMFYLSEKEDSDSPIDSKELAINSNQSQLILILTEKCNLRCEYCIYNDKYPKEMGYSDEEMDFETAKKAVDMYYELHMERVKRGHKRFPVITMYGGEPLLKFDLIKKVMEYAKGLMPDTLFYTTTNGTLLSEKMMDYFINNRIIITFSIDGFKENHDRNRVFVNGMPTFERAFKNIKRLQEKKKEQNIEQIISFNCCFDQYTDVYKVAKFFEEHYDLFNPFFVLYNQINPFDTLYFDWCDEQVKTGKWNFDKNNFKNAMQKIEHELYEAETCDDHFQQVAGPLVMKDFVLSIRNKDGQQQITRNSCIPTSKIAVSPDGTLTLCEKMCKKYPIGTVEKGLDWKAVDGVTEKLVRHFNSDSCKYCPIRTMCEACFMFLDENGRIKPSFCKSKKMAVKKNLESYFAKKEKGFDVMKVYNHTSDLDSVKEMVK**
>**pET28a-TrnD** (His$_6$-**TrnD**)<br>GSSHHHHHHSSGLVPRGSH**MKKYFRLYPYCHLEIGETNSCLYDISSGKMIRVNRENAELLRQCQENVPIESINMDLGILDELIKMNLGTYYANPQFIEPFFETNDTKNRIFGKNNILRQMFILTSTDCNMNCKHCNTDSTVFRKTGCKIWPKSINLNALTQSHWRKILEAFYNLHGEELTFIGGEPFLEFDFIKNIVEIAQEVGISKFSIFTNGSIINDTILNFLMENKIKVYIQIFEVDENKFKAFTNSDIPSIQIIDNIKKLNNHHLDLQLRILITRDNDNNLKKIVNTLQKETNVKDIKIEFLYPKPDNSYYSKKYIPLMYDKKREFSHVNVQKMQFLHQYNPSFFGQITIRRDGKVVPHPMLLTRVIGDLQQDDLFTIINTEEYQEYSTLNK**

EKISKCSTCAYKYNCMDDRVIENFATGDLYGMEYCNF

>pRSFDuet1-TrnC-TrnD (MCS I-His$_6$**-TrnC, MCS II-TrnD)**

MCS I-
GSSHHHHHHSQDP**MSKKRLSMMPFKTDKFSYALDGNTGRVIVADKPTLYIISHFHK
FEKEELLKKTKGKFAELHQDYLTTYNYVSSLINMGMFYLSEKEDSDSPIDSKELAI
NSNQSQLILILTEKCNLRCEYCIYNDKYPKEMGYSDEEMDFETAKKAVDMYYELH
MERVKRGHKRFPVITMYGGEPLLKFDLIKKVMEYAKGLMPDTLFYTTTNGTLLS
EKMMDYFINNRIIITFSIDGFKENHDRNRVFVNGMPTFERAFKNIKRLQEKKKEQN
IEQIISFNCCFDQYTDVYKVAKFFEEHYDLFNPFFVLYNQINPFDTLYFDWCDEQV
KTGKWNFDKNNFKNAMQKIEHELYEAETCDDHFQQVAGPLVMKDFVLSIRNKD
GQQQITRNSCIPTSKIAVSPDGTLTLCEKMCKKYPIGTVEKGLDWKAVDGVTEKL
VRHFNSDSCKYCPIRTMCEACFMFLDENGRIKPSFCKSKKMAVKKNLESYFAKKE
KGFDVMKVYNHTSDLDSVKEMVK**

**MCS II-
KKYFRLYPYCHLEIGETNSCLYDISSGKMIRVNRENAELLRQCQENVPIESINMDL
GILDELIKMNLGTYYANPQFIEPFFETNDTKNRIFGKNNILRQMFILTSTDCNMNC
KHCNTDSTVFRKTGCKIWPKSINLNALTQSHWRKILEAFYNLHGEELTFIGGEPFL
EFDFIKNIVEIAQEVGISKFSIFTNGSIINDTILNFLMENKIKVYIQIFEVDENKFKAFT
NSDIPSIQIIDNIKKLNNHHLDLQLRILITRDNDNNLKKIVNTLQKETNVKDIKIEFL
YPKPDNSYYSKKYIPLMYDKKREFSHVNVQKMQFLHQYNPSFFGQITIRRDGKVV
PHPMLLTRVIGDLQQDDLFTIINTEEYQEYSTLNKEKISKCSTCAYKYNCMDDRVI
ENFATGDLYGMEYCNF**

Table S2 Primers used in this study.

Primers	Oligonucleotide sequence (5' to 3')
Trnα-pCold-F	TCGGTACCCTCGAGGGATCCATGGAAGTTATGAACAATGCTTTAATTAC
Trnα-pCold-R	ACTGCAGGTCGACAAGCTTAACCTAAAGTAAATGCTGTTCCTAC
Trnβ-pCold-F	CGGTACCCTCGAGGGATCCATGGAAGTTTTAAACAAACAAAATG
Trnβ-pCold-R	ACTGCAGGTCGACAAGCTTATAGGAAATAAGATGCAGCTG
TrnC-pRSFDuet-MCS1-F	AATAAGGAGATATACCATGGGCATGTCTAAAAAAAGACTATCAATGATGCC
TrnC-pRSFDuet-MCS1-R	CATTATGCGGCCGCAAGCTTTCATTTTACCATCTCCTTGACAG
His_6-TrnC-pRSFDuet-MCS1-F	CATCACCACAGCCAGGATCCGATGTCTAAAAAAAGACTATCAATGATGCC
TrnC-pRSFDuet-MCS2-F	AAGAAGGAGATATACATATGTCTAAAAAAAGACTATCAATGATGCC
TrnC-pRSFDuet-MCS2-R	GTTTCTTTACCAGACTCGAGTCATTTTACCATCTCCTTGACAG
TrnD-pRSFDuet-MCS2-F	AAGAAGGAGATATACATATGAAAAAATATTTCAGATTGTATCCATATTG
TrnD-pRSFDuet-MCS2-R	GTTTCTTTACCAGACTCGAGTTAAAAGTTACAATATTCCATTCCATAC
TrnD-pET28a-F	TGCCGCGCGGCAGCCATATGAAAAAATATTTCAGATTGTATCCATATTG
TrnD-pET28a-R	GGTGGTGGTGGTGCTCGAGTTAAAAGTTACAATATTCCATTCCATAC
TrnC-C114A-F	AATCACAGCTTATATTAATACTTACTGAGAAGGCTAATTTACGTTGTGAATATTG
TrnC-C114A-R	TCGTTATAAATACAATATTCACAACGTAAATTAGCCTTCTCAGTAAGTATTAATATAAG
TrnC-C114C118A-F	AATACTTACTGAGAAGGCTAATTTACGTGCTGAATATTGTATTTATAACG
TrnC-C114C118A-F	ATATTTGTCGTTATAAATACAATATTCAGCACGTAAATTAGCCTTCTCAG
TrnC-C383A-F	GGTCAACAACAAATTACCAGAAATAGTGCTATTCCTACTAGTAAAATAGC
TrnC-C383A-R	GGAGAAACAGCTATTTTACTAGTAGGAATAGCACTATTTCTGGTAATTTG
TrnC-C400A-F	CTGTTTCTCCTGATGGTACTCTAACATTGGCTGAAAAAATGTGTAAAAAG
TrnC-C400A-R	ATTGGATACTTTTTACACATTTTTTCAGCCAATGTTAGAGTACCATCAG
TrnC-C404A-F	GGTACTCTAACATTGTGTGAAAAAATGGCTAAAAAGTATCCAATAG
TrnC-C404A-R	AACAGTGCCTATTGGATACTTTTTAGCCATTTTTTCACACAATG

TrnC-C437A-F	TAGTTCGGCATTTTAATAGTGACTCAGCTAAATACTGTCCGATTAG
TrnC-C437A-R	ACATTGTTCTAATCGGACAGTATTTAGCTGAGTCACTATTAAAATG
TrnC-C437C440A-F	CATTTTAATAGTGACTCAGCTAAATACGCTCCGATTAGAACAATGTGTG
TrnC-C437C440A-R	GCACGCTTCACACATTGTTCTAATCGGAGCGTATTTAGCTGAGTCAC
TrnC-C446A-F	GTAAATACTGTCCGATTAGAACAATGGCTGAAGCGTGCTTTATGTTC
TrnC-C446A-R	TCATCCAGGAACATAAAGCACGCTTCAGCCATTGTTCTAATCGGAC
TrnC-C449A-F	TGTCCGATTAGAACAATGTGTGAAGCGGCATTTATGTTCCTGGATG
TrnC-C449A-R	ACCATTTTCATCCAGGAACATAAATGCCGCTTCACACATTGTTC
TrnC-C464A-F	AAATGGTCGAATTAAGCCTTCATTTGCCAAATCTAAGAAGATG
TrnC-C464A-R	CTTTACTGCCATCTTCTTAGATTTGGCAAATGAAGGCTTAATTC
TrnC-R370AK372A-F	TGAAAGATTTTGTTCTTTCAATAGCTAATGCTGATGGTCAACAACAAATTAC
TrnC-R370AK372A-R	TATTTCTGGTAATTTGTTGTTGACCATCAGCATTAGCTATTGAAAGAAC
TrnC-ΔRRE-F	GCCGCGCGGCAGCCATAAAGAAGATTCTGATTCACCGATTGATTCAAAGGAACTTGC
TrnC-ΔRRE-R	TCAATCGGTGAATCAGAATCTTCTTTATGGCTGCCGCGCGGCACCAG
TrnD-C109A-F	AGACAAATGTTCATTTTAACTTCCACAGATGCTAACATGAATTGTAAGCACTG
TrnD-C109A-R	CCGTATTACAGTGCTTACAATTCATGTTAGCATCTGTGGAAGTTAAAATGAAC
TrnD-C109C113A-F	TTAACTTCCACAGATGCTAACATGAATGCTAAGCACTGTAATACGGATTC
TrnD-C109C113A-R	AAACAGTTGAATCCGTATTACAGTGCTTAGCATTCATGTTAGCATCTGTG
TrnD-C383A-F	TCAATAAAGAGAAAATATCCAAAGCTTCAACATGCGCATACAAG
TrnD-C383A-R	TACAATTATACTTGTATGCGCATGTTGAAGCTTTGGATATTTTCTC
TrnD-C383C386A-F	TCAATAAAGAGAAAATATCCAAAGCTTCAACAGCCGCATACAAG
TrnD-C383C386A-R	TACAATTATACTTGTATGCGGCTGTTGAAGCTTTGGATATTTTCTC
TrnD-C392A-F	TTCAACATGCGCATACAAGTATAATGCTATGGATGACCGAGTAATTG
TrnD-C392A-R	AGCAAAATTTTCAATTACTCGGTCATCCATAGCATTATACTTGTATGCG
TrnD-C412A-F	CTACCGGTGATTTGTATGGAATGGAATATGCTAACTTTTAACTCGAGTCTGG
TrnD-C412A-R	TTTCTTTACCAGACTCGAGTTAAAAGTTAGCATATTCCATTCCATACAAATC
TrnD-E79A-F	TACGTATTATGCTAATCCACAATTCATTGCTCCTTTTTTTGAAACTAATG
TrnD-E79A-R	TTTTCGTATCATTAGTTTCAAAAAAAGGAGCAATGAATTGTGGATTAGC
TrnD-ΔRRE-F	CCGCGCGGCAGCCATAATGATACGAAAAACAGAATATTCGGTAAGAATAATATATT

	AAG
TrnD-ΔRRE-R	CGAATATTCTGTTTTTCGTATCATTATGGCTGCCGCGCGGCACCAG

References

1. M. Strohalm, M. Hassman, B. Kosata, M. Kodícek, mMass data miner: an open source alternative for mass spectrometric data analysis. *Rapid Commun. Mass Spectrom.* **22**, 905-908 (2008).
2. M. C. Rea, C. S. Sit, E. Clayton, P. M. O'Connor, R. M. Whittal, J. Zheng, J. C. Vederas, R. P. Ross, C. Hill, Thuricin CD, a posttranslationally modified bacteriocin with a narrow spectrum of activity against Clostridium difficile. *Proc. Natl. Acad. Sci. U. S. A.* **107**, 9352-9357 (2010).
3. S. Jang, J. A. Imlay, Hydrogen peroxide inactivates the Escherichia coli Isc iron-sulphur assembly system, and OxyR induces the Suf system to compensate. *Mol Microbiol* **78**, 1448-1467 (2010).
4. N. D. Lanz, T. L. Grove, C. B. Gogonea, K. H. Lee, C. Krebs, S. J. Booker, RlmN and AtsB as models for the overproduction and characterization of radical SAM proteins. *Methods Enzymol* **516**, 125-152 (2012).
5. W. W. Fish, Rapid colorimetric micro-method for the quantitation of complexed iron in biological samples. *Methods Enzymol.* **158**, 357-364 (1988).
6. H. Beinert, Semi-micro methods for analysis of labile sulfide and of labile sulfide plus sulfane sulfur in unusually stable iron-sulfur proteins. *Anal. Biochem.* **131**, 373-378 (1983).
7. W. Q. Liu, P. Amara, J. M. Mouesca, X. Ji, O. Renoux, L. Martin, C. Zhang, Q. Zhang, Y. Nicolet, 1,2-Diol Dehydration by the Radical SAM Enzyme AprD4: A Matter of Proton Circulation and Substrate Flexibility. *J. Am. Chem. Soc.* **140**, 1365-1371 (2018).
8. E. Richard, O. N. Michael, P. Alexander, A. Natasha, S. Andrew, G. Tim, Ž. Augustin, B. Russ, B. Sam, Y. Jason, R. Olaf, B. Sebastian, Z. Michal, B. Alex, P. Anna, C. Andrew, T. Kathryn, J. Rishub, C. Ellen, K. Pushmeet, J. John, H. Demis, Protein complex prediction with AlphaFold-Multimer. *bioRxiv*, 2021.2010.2004.463034 (2022).
9. M. Mirdita, K. Schütze, Y. Moriwaki, L. Heo, S. Ovchinnikov, M. Steinegger, ColabFold: making protein folding accessible to all. *Nature Methods* **19**, 679-682 (2022).
10. S. Yuan, H. C. S. Chan, Z. Hu, Using PyMOL as a platform for computational drug design. *Wiley Interdisciplinary Reviews: Computational Molecular Science*, e1298-n/a (2017).
11. J. I. Tietz, C. J. Schwalen, P. S. Patel, T. Maxson, P. M. Blair, H. C. Tai, U. I. Zakai, D. A. Mitchell, A new genome-mining tool redefines the lasso peptide biosynthetic landscape. *Nat Chem Biol* **13**, 470-478 (2017).
12. G. A. Hudson, B. J. Burkhart, A. J. DiCaprio, C. J. Schwalen, B. Kille, T. V. Pogorelov, D. A. Mitchell, Bioinformatic Mapping of Radical S-Adenosylmethionine-Dependent Ribosomally Synthesized and Post-Translationally Modified Peptides Identifies New Cα, Cβ, and Cγ-Linked Thioether-Containing Peptides. *Journal of the American Chemical Society* **141**, 8228-8238 (2019).
13. C. L. M. Gilchrist, Y.-H. Chooi, clinker & clustermap.js: automatic generation of gene cluster comparison figures. *Bioinformatics* **37**, 2473-2475 (2021).
14. K. Katoh, D. M. Standley, MAFFT multiple sequence alignment software version 7: improvements in performance and usability. *Mol Biol Evol* **30**, 772-780 (2013).
15. T. L. Grove, P. M. Himes, S. Hwang, H. Yumerefendi, J. B. Bonanno, B. Kuhlman, S. C. Almo, A. A. Bowers, Structural Insights into Thioether Bond Formation in the Biosynthesis of Sactipeptides. *J Am Chem Soc* **139**, 11734-11744 (2017).
16. K. M. Davis, K. R. Schramma, W. A. Hansen, J. P. Bacik, S. D. Khare, M. R. Seyedsayamdost, N. Ando, Structures of the peptide-modifying radical SAM enzyme SuiB elucidate the basis of substrate recognition.

Proc Natl Acad Sci U S A **114**, 10420-10425 (2017).
